# Access to Anti-Biofilm Compounds from Endolichenic Fungi Using a Bioguided Networking Screening

**DOI:** 10.3390/jof8101012

**Published:** 2022-09-27

**Authors:** Seinde Toure, Marion Millot, Lucie Ory, Catherine Roullier, Zineb Khaldi, Valentin Pichon, Marion Girardot, Christine Imbert, Lengo Mambu

**Affiliations:** 1 Laboratoire PEIRENE, University Limoges, UR 22722, F-87000 Limoges, France; 2 Institut des Substances et Organismes de la Mer (ISOMer), Nantes Université, UR 2160, F-44000 Nantes, France; 3 Laboratoire Ecologie et Biologie des Interactions (EBI), University Poitiers, UMR CNRS 7267, F-86000 Poitiers, France

**Keywords:** endolichenic fungi, *Candida* biofilms, metabolomic, fatty acids

## Abstract

Endolichenic microorganisms represent a new source of bioactive natural compounds. Lichens, resulting from a symbiotic association between algae or cyanobacteria and fungi, constitute an original ecological niche for these microorganisms. Endolichenic fungi inhabiting inside the lichen thallus have been isolated and characterized. By cultivation on three different culture media, endolichenic fungi gave rise to a wide diversity of bioactive metabolites. A total of 38 extracts were screened for their anti-maturation effect on *Candida albicans* biofilms. The 10 most active ones, inducing at least 50% inhibition, were tested against 24 h preformed biofilms of *C. albicans*, using a reference strain and clinical isolates. The global molecular network was associated to bioactivity data in order to identify and priorize active natural product families. The MS-targeted isolation led to the identification of new oxygenated fatty acid in *Preussia persica* endowed with an interesting anti-biofilm activity against *C. albicans* yeasts.

## 1. Introduction

Over the past two decades, the exploration of endophytic world has exploded, and many original natural compounds have emerged. In the same way as plants, lichens constitute a suitable environment for the development of microorganisms. A wide diversity of bacteria and fungi has been identified as epilichenic or endolichenic symptomless microorganisms. The epilichenic or endolichenic fungi are distinct from mycobionts and from lichenicolous fungi. The majority of species belongs to Pezizomycotina and are distributed in seven orders (Hypocreales, Sordariales, Eurotiales, Xylariales, Leotiales, Chaetothyriales, Phaesporales) [1,2,3]. Investigations on endolichenic fungi over the last 10 years revealed potential new bioactive secondary metabolites [1]. Phaeosphaerins, nodulisporiviridins, conioxepinols and coniothiepinols are among novel skeletons that have been characterized in such organisms [1]. Secondary metabolites produced by endophytes and endolichenic fungi have been reported for varied biological activities such as antiproliferative, antimicrobial, or anti-inflammatory [1]. In the race for alternatives to current antimicrobials, metabolites from fungal endophytes and endolichenic fungi are thus a hidden reservoir for discovering the next generation of biopharmaceuticals [1,2]. Certain *Candida* species are major human fungal pathogens that cause both mucosal and deep tissue infections [4]. In the majority of infections, these microorganisms form structured, coordinated and functional communities called biofilms. In biofilms, fungal hyphae, when they exist depending on the species, and yeast-forms are embedded in a self-created extracellular matrix particularly resistant. Seventeen *Candida* species are known to be involved in human infections but 90% of invasive infections are caused by *C. albicans*, *C. glabrata*, *C. parapsilosis*, *C. tropicalis* and *C. krusei* [4]. Interaction between cells and substrates make sessile microorganisms complex and more robust and resistant to conventional treatment [5,6,7]. The currently available antifungal drugs exert their activity either with actions on the fungal membrane (polyene and azole antifungals) or by disrupting the fungal cell wall (echinocandins). Among these drugs, only amphotericin B lipid formulations and echinocandins have demonstrated in vitro activity against *C. albicans* biofilms [8,9,10,11]. Interestingly, echinocandins were obtained by fermentation of filamentous fungi such as *Aspergillus* sp. and their hemisynthesized derivatives (caspofungin, micafungin and anidulafungin) are clinically used. Thus, natural kingdom (plants, fungi, algae, or lichens) is progressively explored and studied to find new drug candidates for biofilm eradication. Among fungal metabolites, myriocin, first isolated from *Myriococcum albomyces*, completely prevented the formation of *C. albicans* biofilms [12]. Also, shearinines D and E, two alkaloids isolated from *Penicillium* sp. inhibited the formation of *C. albicans* biofilms [13]. Some lichen extracts have also been previously studied for their anti-biofilm activity against *C. albicans* biofilms [14]. *Evernia prunastri* and *Ramalina fastigiata* acetonic extracts were pointed out as the most active lichens. Their anti-biofilm activity was attributed to the main metabolite: evernic acid [15]. To deeply explore the contribution of lichen associated microorganisms in the biological activity of lichen extracts, endolichenic fungi previously isolated from these lichens were cultivated and extracted in order to evaluate their ability to prevent biofilm maturation phase or to eradicate an already formed biofilm. In addition, this study aimed to increase knowledge on chemical composition of endolichenic fungi, especially those possibly involved in biological activities.

## 2. Materials and Methods

### 2.1. Chemicals

Acetone, Acetonitrile HPLC grade, cyclohexane, ethyl acetate, dichloromethane, toluene, methanol (all with purity ≥ 99.8%) were purchased from Carlo Erba. Methanol (HPLC grade, ≥99.8%), chloroform (≥99.2%), and formic acid (≥99.3%) were purchased from VWR International.

### 2.2. General Experimental Procedures

HPLC analyzes were performed on an Alliance Waters 2690 using a reversed-phase EC Nucleodur C-18 Tec column (250 × 4.6 mm, 10 µm) coupled with a Photodiod Array detector Waters 996 and a DEDL-TL SEDEX85 Sedere. HR-MS analyses have been performed in the ICOA platform on a Brucker maxis II UHR-qTOF MS. NMR analyzes have been performed at the BISCEm plateform on a Brucker Avance III HD, Brucker. GC-MS analyses have been performed on a QP20210 SE with QP Series on an Optima 5MS (30 m × 0.25 mm × 0.25 µm) column. Data acquisition have been made with the LabSolution LC/GC software. MPLC was carried out using the Büchi pump model C-605, C-615 with silica gel 60, 40–63 µm, Merk^®^.

### 2.3. Fungi and Culturing Conditions

Endolichenic fungi have been obtained from four lichens (*Evernia prunastri* (EP), *Ramalina fastigiata* (RF), *Nephroma laevigatum* (NL) and *Pleurosticta acetabulum* (PA)) [16]. Eleven fungal species including 14 strains have been studied: *Anthostomella pinea*, *Biscogniauxia mediterranea* (genotype 1 and 2), *Biscogniauxia nummularia* (genotype 1 and 2), *Coniochaeta decumbens*, *C. hoffmannii*, *C. lignicola*, *Fusarium avenaceum*, *Peziza varia*, *Plectania* sp, *Preussia persica* (Genotype 1 and 2), and *Sordaria fimicola* (Supplementary Data, Table S1). Mycelia stored at −80 °C were pre-cultured on Potato Dextrose Agar (PDA) medium (Roth). In a second step, mycelia were cultivated on three different media: PDA, Sabouraud (SAB) and Malt Extract Agar (MEA) (Roth). Ten agar plugs (9 mm diameter) were placed in a 90 mm Petri dish containing 20 mL of each medium were inoculated with fungal strains. Fungi grew 21 days in a dark room, without agitation at 25 °C before extraction.

### 2.4. Extraction and Isolation of Compound 1

Small scale culture and extraction: medium with fungus was cut into small pieces and extracted in an erlenmeyer with 200 mL of EtOAc under stirring for 24 h. After filtration, the resulted filtrate was washed twice with 200 mL of distilled water, and dehydrated with Na_2_SO_3_. The organic phase was concentrated under vacuum to give 38 crude extracts.

Large scale culture and extraction: *P. persica* G2 was cultivated for 21 days at 25 °C on MEA medium (10 L) in Petri dishes (120 mm) with 50 mL in each. A total of 200 Petri dishes were used. The medium containing fungal hyphae was then extracted with 10 L of EtOAc twice. Gelose was stirred with a Heidoplph stirrer Hei-TORQUE 100. The resulting filtrate was concentrated under vacuum until 150 mL and then washed with distilled water (150 mL). After filtration and drying on Na_2_SO_3_, the organic layer was concentrated and yielded to 560 mg of an EtOAc extract. The crude extract was submitted to a silica gel (40 g, 0.040–0.063 mm) with an elution gradient C_6_H_5_-EtOAc 80:20 to 0:100. Fifteen fractions were obtained. The HPLC-DAD-DEDL of fraction F11 (13.0 mg) show a single signal detected by the DEDL which corresponding to a pure compound. The peak has been analyzed by NMR and HR-MS and identified as 8Z-5,6-epoxy-4-hydroxy-octadec-8-enoic acid. A second large scale culture has been carried out in the same conditions leading to a crude EtOAc extract. After water washing and drying, the remaining organic extract (400 mg) was treated with a mixture of *n*-hexane, water, and methanol (20:2:18). This liquid/liquid extraction led to a hexanic extract E1 (210 mg) and a hydro-methanolic extract E2 (190 mg)

Compound 1: [α]^26^_D_ = −10 (c 0.2, CH_2_Cl_2_). ESI-HR-MS [M + H]^+^ at *m/z* 313.2372 (calc. 313.2373) C_18_H_32_O_4_. NMR ^1^H (Acetone-*d*_6_, 500 MHz, δ, *J* in Hz) 0.89 (t, *J* = 6.9, H-18), 1.35 (m, H11 to H-17), 1.60 (m, H-3), 2.05 (dt, *J* = 2.2, 4.4, H-10), 2.29 (t, *J* = 7.4, H-2), 2.37 (bdd, *J* = 5.6, 10.4, H-7), 2.75 (dd, *J* = 4.1, 8.2, H-5), 2.88 (ddd, *J* = 4.1, 5.6, 9.7, H-6), 3.44 (td, 4.4, 8.2, H-4), 5.50 (bdd, *J* = 5.9, 10.8, H-8), 5.52 (bdd, *J* = 5.9, 10.8, H-9) RMN ^13^C (Acetone-*d*_6_, 130 MHz): 14.4 (C-18), 25.8 (C-3), 27.1 (C-7), 28.0 (C-10), 30.0–30.1 (C11-C17), 34.3 (C-2), 57.0 (C-6), 60.2 (C-5), 69.2 (C-4), 125.9 (C-8), 132.9 (C-9), 174.8 (C-1) (Supplementary materials, Figures S22–S30)

### 2.5. Anti-Maturation and Anti-Biofilm Assays

Stock solutions of fungal extracts were prepared in DMSO at 10 mg/mL and shaken. *C. albicans* ATCC^®^ 28367™ strain was purchased from the American Type Culture Collection. The three clinical strains were provided by the laboratory of parasitology and medical mycology of the hospital of Poitiers (France) and were isolated from catheters from patients. The anti-maturation and anti-biofilm activities were assessed as previously described [14]. Serial twofold dilutions of each stock solution were prepared in Yeast Nitrogen Base medium (Difco, Detroit, MI, USA), supplemented with 50 mM glucose (Sigma, St. Louis, MO, USA) (YNB-Glc) over the final range 200 μg/mL to 0.097 μg/mL for the anti-maturation test or at 200, 100 and 50 μg/mL for the anti-biofilm tests (final DMSO concentrations did not exceed 2% of the overall volume in wells). *C. albicans* yeasts were first grew for 24 h on Sabouraud agar slants. Then, several loopfuls of this culture were carefully transferred to 30 mL of YNB-Glc and incubated overnight at 37 °C without shaking. Obtained yeasts were washed twice in 0.1 M phosphate-buffered saline (PBS, pH 7.2) and adjusted to 1 × 10^7^ blastospores/mL in YNB-Glc.

Untreated 96-well tissue culture plates were filled with 200 µL of yeast suspension at 1.10^7^ cells/mL. After 2 h of incubation at 37 °C for the anti-maturation tests or 24 h of incubation at 37 °C for the anti-biofilm tests, the non-adherent yeasts were removed by washing with PBS. Then, 250 μL of YNB-Glc and 50 μL of each dilution of fungi extracts were added to each well. Some wells were reserved for controls: medium control, non-treated yeasts (negative control) and yeasts treated by DMSO 2%. The plates were incubated for 24 h and/or 48 h at 37 °C. Then spent media and free-floating microorganisms were then removed by aspiration. Wells were washed twice with PBS and observed under inverted optical microscope (IX51^®^ inverted microscope, Olympus Corporation, Tokyo, Japan) prior to biofilm quantification using a previously described metabolic assay based on the reduction of a tetrazolium salt (XTT) [8,17]. Briefly, 300 mg/L XTT (MerckKGaA, Darmstadt, Germany) and 0.13 mM menadione (Merck KGaA, Darmstadt, Germany) were added to 200 μL of PBS in each well. Plates were incubated for 3 h at 37 °C without shaking, then gently agitated and XTT formazan was measured colorimetrically at 492 nm (microplate reader LP400; Sanofi Diagnostics Pasteur, Marnes-la-coquette, France). The percentage inhibition was calculated from the absorbance values compared to the mean absorbance value obtained for untreated wells (100%). All anti-maturation and anti-biofilm tests were performed at least in triplicate in at least two separate experiments.

### 2.6. Statistical Analysis

Statistical analysis was performed by Mann-Whitney test to determine differences with non-treated control.

The principal component analysis was performed using the online MetaboAnalyst platform version 4.0 [18], after Pareto scaling and cube root transformation of the data.

### 2.7. LC-HRMS Analyses

LC-HRMS analyses were performed using an Ultimate 3000 RSLC system (Dionex, Germering, BY, Germany) coupled to a maXis Q-TOF (Bruker, Bremen, Germany). The separation was achieved on a Luna Omega C18 (2.1 × 150 mm) with a particle size of 1.6 µm (Phenomenex, Le Pecq, France) fitted with a C18 SecurityGuard Ultra (2.1 mm) guard filter (Phenomenex). The column was thermostated at 40 °C and a solvent gradient using water + 0.1% formic acid (solvent A) and acetonitrile + 0.08% formic acid (solvent B) at 500 µL/min was applied as follows: 3% B from 0 to 0.1 min, 50% B at 10 min, 100% at 15 min for 5 min, 3% B at 20.1 min up to 25 min. The crude extracts were reconstituted at 5 mg/mL with methanol and 0.5 µL were injected. Mass experiments were recorded with an ESI ion source in negative mode with a capillary voltage set at 4 kV, nebulizing gas at 2 bar and 9 L/min for drying gas heated at 200 °C. MS/MS analyses were acquired using of Control 3.4 (Bruker, Bremen, Germany) in the 50–1650 *m/z* range by selection of 3 precursors between 135 and 1000 *m/z*; ions were excluded after one selection and released after 0.2 min except if their intensity was superior to 4-time the previous one. Summation times were 400 ms for both MS and MS/MS spectra, leading to 1.6 s per cycle. CID was performed at 25 eV using nitrogen as collision gas. Data were converted into mzXML using CompassXport 3.0.13 (Bruker, Bremen, Germany) after lock mass recalibration in DataAnalysis 4.4 software (Bruker, Bremen, Germany).

### 2.8. Data Treatment

Peak picking. HPLC-ESI(-)HRMS/MS profiles were exported under *.mzXML format. These raw data were first submitted to peak picking by using MZmine2 software (version 2.53). Peak detection in negative mode was achieved with the “mass detection” algorithm with a noise level of “5E2” in centroid mode. Then, chromatograms were built for all detected ions with the “ADAP chromatogram builder” algorithm with a min group size scan of 2, group intensity threshold of 500, min highest intensity of 500 and *m/z* tolerance of 0.02 or 0 ppm. Peak deconvolution was applied to generate chromatograms with the “Wavelets” algorithm with following parameters: S/N threshold of 5, intensity window SN as S/N estimator, min feature height of 500, coefficient/area threshold of 35, peak duration range from 0.01 to 5, and RT wavelet range from 0.01 to 0.20. Isotope removal was carried out with the isotopic peaks grouper with the following parameters: *m/z* tolerance of 0.005, RT tolerance of 0.1 min, maximum charge of 3 and the most representative ion kept was the most intense. Duplicates were removed based on the “New average method” filter by applying a 0.005 *m/z* tolerance and a 0.1 min RT tolerance. A peak list rows filter was applied to keep only peaks in the 0.9–21 min RT range, with an intensity higher than 1E3. Alignment was then performed with a *m/z* tolerance of 0.005 and a RT tolerance of 0.1 min, with the same weight for both parameters. Finally, a last step of gapfilling was performed using the “Peak finder” module, with an intensity tolerance of 50%, *m/z* tolerance of 0.005 and a RT tolerance of 0.1 min, yielding a combined dataset. The features detected from blank MeOH and non-inoculated culture medium samples (BM) were removed from the generated matrix to focus on the features really corresponding to the fungus production. The first data matrix combining the results from all strains and used for PCA, gathered 13482 peaks with their corresponding peak areas in the different samples. After removal of *Coniochaeta* and *Biscognauxia* samples, the matrix retained 7397 peaks. Then, separated export for each genus from MZmine generated 4 *.mgf files with MS2 data for peaks and a *.csv file with the area peak integration values for each peak in each sample group. The files generated gathered 3468 peaks with corresponding MS2 data and peak areas for *Coniochaeta* samples, 2779 peaks for *Biscogniauxia* samples, 1225 peaks for *Preussia* samples and 1245 peaks for *Anthostomella* samples.

Molecular network. Molecular network was built using both Metgem and the GNPS platform. The following parameters were applied for MetGem: for cosine score computing, MS1 data was ticked, *m/z* tolerance was set to 0.02 Da and minimum matched peaks to 4; filtering kept peaks outside the ±17 Th window, above 0% of maximum and each peak in top 6 in the 50 Th window; network visualization allowed a maximum neighbor number (topK) of 10, a minimal cosine score of 0.65 and a maximum connected component size of 100. t-SNE visualization used the following parameters: at least 1 cosine score above 0.7, number of iterations of 1000, perplexity of 6, learning rate of 200, early exaggeration of 12 and the Barnes-Hut approximation with an angle of 0.5. The following parameters were applied for GNPS: precursor ion mass tolerance 0.02 Da; Fragment ion mass tolerance 0.02 Da; Min pairs cos 0.65; Network TopK 10; Maximum connected component size: 100; Minimum matched fragment ions 4; minimum cluster size 2; Run MS cluster No. Library search was performed on all the available spectral libraries from GNPS with the following parameters: library search min matched peaks 4; score threshold 0.7: search for analogs with a maximum mass difference of 100. Default filtering options were used.

Biochemometrics. To assign a bioactivity score to the different nodes presented on the network, the FiBiCo script was applied on R to the peak list matrix additioned with bioactivity values (adherence inhibition percentages and activity group) [19]. The scores obtained from F-PCA, Spearman and PLS-DA were summed to obtain the final score presented on molecular networks. For *Coniochaeta* samples, activity groups were defined according to 48 h adherence inhibition at 100 µg/mL, with <40% inhibition in group 1 (inactive), 40–50% inhibition in group 2 (moderate activity) and >50% inhibition in group 3 (active group). For *Biscogniauxia* samples, activity groups were defined according to 48 h adherence inhibition at 200 µg/mL, with <30% inhibition in group 1 (inactive), 30–50% inhibition in group 2 (moderate activity) and >50% inhibition in group 3 (active group). For *Preussia* samples, activity groups were defined according to 48 h adherence inhibition at 50 µg/mL, with <40% inhibition in group 1 (inactive), 40–50% inhibition in group 2 (moderate activity) and >50% inhibition in group 3 (active group).

## 3. Results

### 3.1. Extracts Library

In a previous study, lichen-associated fungi of *E. prunastri*, *R. fastigiata*, *Pleurosticta acetabulum* and *Nephroma laevigatum* were studied and isolated by culture on Potato Dextrose Agar (PDA) and Malt Extract Agar (MEA) media [16,20]. After molecular identification, fungal isolates were stored at −80 °C. Among the twenty endolichenic species properly identified, eleven were successfully cultivated after storage. For three species (*B. nummularia*, *B. mediterranea* and *P. persica*), two different genotypes were selected in order to observe metabolites variations. Thus, 14 fungal strains selected were cultivated on three different culture media (PDA, MEA, and SAB). After 21 days of culture at room temperature, fungi can be distributed into three groups related to their speed of growth: fast growing fungi (*Biscaugniauxia*, *Fusarium*, *Plectania* and *Sordaria* species); moderate growth fungi (*Coniochaeta* and *Peziza* species) and slow growth fungi (*A. pinea* and *P. persica*). A longer time of culture have been applied for the fungi of the third group. Finally, 40 EtOAc extracts were obtained as two strains (corresponding to *B. mediterranea* (genotype 2) and *B. nummularia* (genotype 2) not having grown on MEA medium. Thirty-eight extracts have been obtained with sufficient quantity for biological evaluation on biofilm.

### 3.2. Anti-Biofilm Screening Results

The inhibition level of the biofilm maturation phase induced by extracts at 100 µg/mL was taken into account for selecting the best anti-biofilm extracts candidates. The overall trend showed a better activity after 48 h of contact between the extract and the biofilm. This extended and delayed effect has already been observed on other extracts [14]. Overall, after a contact of 48 h at 100 µg/mL, 10 of the 38 studied extracts reduced significantly the biofilm maturation phase by at least 50% (*p* ≤ 0.0002) (Table 1).

**Figure 1:**
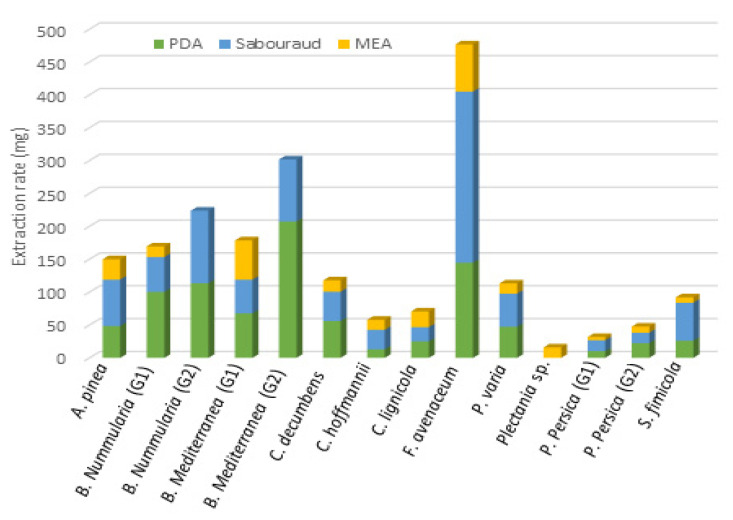
Total quantity of crude fungal extracts obtained from the three culture media.

Three endolichenic fungi extracts showed anti-maturation abilities regardless of the composition of the culture medium, with inhibition ranging between 48% and 71%: *A. pinea* (AP), *C. lignicola* (CL) and *P. persica* G2 (PP). The extracts obtained from *C. decumbens* and *Plectania* sp. species grown in MEA medium displayed significant inhibitory activities, up to 65% (*p* = 0.0002). It could be noticed that, when the fungal growth is more difficult (for example in MEA medium, see Figure 1), the activity towards *Candida* biofilms is better. Two extracts obtained from *Plectania* sp. were not evaluated for their biological activity due to the low extraction rate obtained.

The ten extracts reducing the maturation phase by at least 50% after a contact of 48 h (Table 1) were then tested for their ability to reduce a preformed 24 h-biofilm (Table 2).

The inhibition percentages obtained after a contact of 24 h and 48 h with each extract at 100 µg/mL were indicated in Table 2. Only four extracts, obtained from two fungal species, were found to be capable of both inhibiting biofilm maturation and reducing a pre-formed biofilm after 48 h (*p* < 0.0002). Indeed, it is generally more difficult to disrupt a biofilm that has already formed than to prevent it from forming. All extracts of *A. pinea* were active, inhibiting significantly preformed biofilms by more than 50% (48 h, *p* < 0.0001). Curiously, only the MEA extract of *P. persica* G2 was active (almost 68% inhibition at 48 h, *p* < 0.0001) whereas extracts prepared using other culture media were devoid of activity. Interestingly, three of the four active extracts (AP_PDA, AP_MEA and PP_2_MEA) displayed significant early and persistent anti-biofilm activities, as inhibition percentages were at least 50.7% independently of the contact duration (*p* < 0.0001). Based on this second screening, the four most efficient extracts were deeper investigated to evaluate the strain dependence of their activity. To do that, their activity was evaluated against three clinical isolates of *C. albicans* (Table 3). The *A. pinea* extracts showed significant inhibition percentages above or equal to 50% (*p* < 0.0001) with only one or two clinical strains demonstrating a strain-dependent activity. *Preussia persica* MEA extract was the most promising, being the only one showing a non-strain-dependent activity and significantly inhibiting biofilms of the three clinical isolates by at least 57% (*p* < 0.0001).

In order to link the anti-biofilm activity observed in relation to chemical composition of extracts, extracts were submitted to an LC-MS^2^ analysis.

### 3.3. First Insight into Endolichenic Fungi Metabolomes

**Figure 2:**
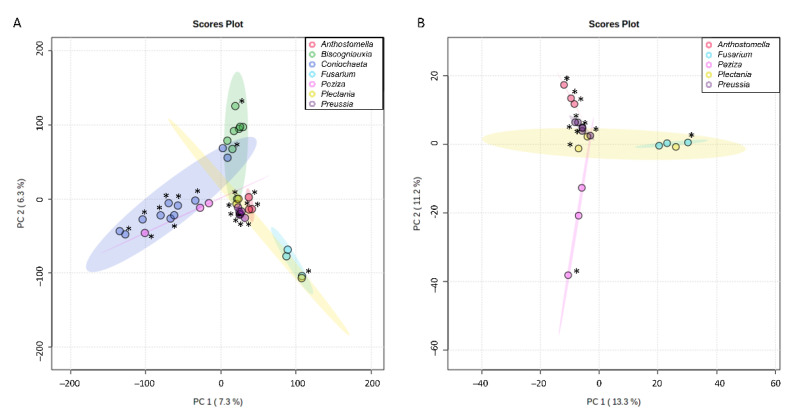
Principal component analysis score plots for the 38 endolichenic fungal extracts investigated based on HPLC-MS/MS data figuring the 2 first principal components for the totality of the extracts (**A**) or after removal of signals from *Coniochaeta* and *Biscognauxia* extracts (**B**). Colors were attributed to the different fungal genus investigated in the present study (*Anthostomella* (red), *Biscogniauxia* (green), *Coniochaeta* (blue), *Fusarium* (cyan), *Peziza* (pink), *Plectania* (yellow), *Preussia* (purple)). Active extracts (*) inducing more than 50% adherence inhibition at 50, 100 or 200 µg/mL in 48 h are highlighted. They correspond to AP_SAB (*Anthostomella pinea*), AP_PDA (*A. pinea*), AP_MEA (*A. pinea*), CL_SAB (*C. lignicola*), CL_PDA (*C. lignicola*), CL_MEA (*C. lignicola*), PP_1_PDA (*P. persica*), PP_1_SAB (*P. persica*), PV_MEA (*P. varia*), PSP_MEA (*Plectania* sp.), CD_MEA, (*Coniochaeta decumbens*), CH_PDA (*Coniochaeta hoffmannii*), CH_MEA (*C. hoffmannii*), CH_SAB (*C. hoffmanii*), PP_2_MEA (*Preussia persica*), PP_2_PDA (*P. persica*), PP_2_SAB (*P. persica*), FA_PDA (*Fusarium avenaceum*), BM_1_PDA (*Biscogniauxia mediterranea*), BM_1_SAB (*Biscogniauxia mediterranea*).

All extracts obtained after culture of endolichenic fungi were analyzed using HPLC-ESI (-)-MS/MS. As a first approach, their metabolomes were investigated using unsupervised multivariate analysis. The principal component analysis score plot (Figure 2A) revealed that the extracts mainly clustered according to the fungal genus, which showed the consistency between chemical patterns on the three culture media and taxonomy. Even after removing signals from *Coniochaeta* and *Biscogniauxia* extracts, which were the best samples separated against principal components 1 and 2 (Figure 2A), most of the variability (with PC1 accounting for 13.3% and PC2 11.2%) remained attributable to fungal genus for other samples (Figure 2B). Indeed, *Anthostomella* and *Preussia* samples, which were very close in the first Principal Component Analysis (PCA), were better separated in the second one. *Plectania* samples were the only ones for which important chemical difference has been observed when the culture media changed.

According to this first insight into endolichenic fungal metabolomes, it was decided to treat the data from samples by genus for further investigation.

### 3.4. Research for Active Compounds by Biochemometrics and Molecular Networking

To highlight the compounds related to the activity observed, the recently described FiBiCo script was applied to the different features recovered from peak picking analysis [19]. In fact, peak areas for each feature were compared together and analyzed in correlation with the anti-maturation activity observed in the different extracts. Each feature then obtained a score, which value was in relation with its potential responsibility in the activity observed. The higher the score, the most chances it was related to the activity. These scores were then mapped on the molecular network obtained for the different genera investigated, in order to highlight families of compounds of biological interest. Molecular networks [21] initially applied to natural products by Dorrestein’s lab, are based on MS/MS fragmentation similarity between spectra among chromatograms. It allows to visually interpret multiple data by clustering similar fragmentation patterns, mostly corresponding to similar chemical patterns. Moreover, searching for the different MS/MS spectra acquired in spectra libraries (GNPS, ISDB, in-house, etc.) [22,23] can also help for compound identification. In the present case, it allowed us to visualize the chemical diversity produced by the endolichenic fungi, link close chemical structures together and highlight the nodes corresponding to the most probable active compounds. By defining the size of the nodes of the network in relation to this score, we were able to highlight the presence of several nodes of interest in several clusters (network obtained with the following parameters: minimum of 4 common fragments, cosine > 0.7).

#### 3.4.1. Investigation of Extracts from *Coniochaeta* Genus

**Figure 3:**
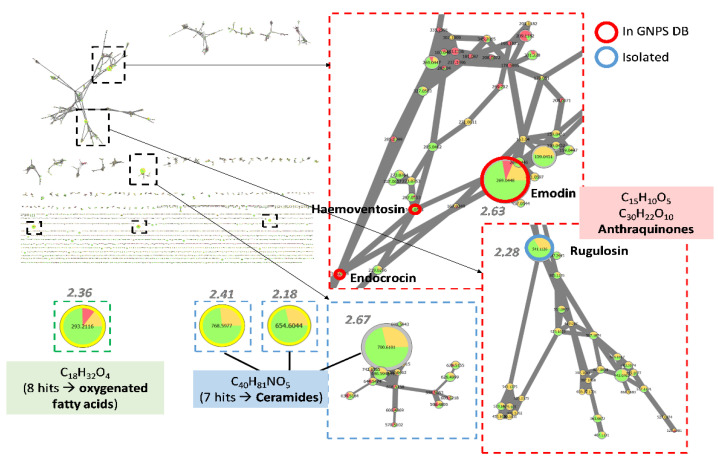
Feature-based molecular network corresponding to *Coniochaeta* extracts. Nodes are filled with pie charts representing the mean peak areas for each feature in the 3 defined groups of activity (anti-maturation activity at 100 µg/mL in 48 h): low (red) for 0–40%, medium (orange) for 40–60% and high (green) for 60–100%. Node sizes are related to FiBiCo scores indicated in grey, highlighting compounds mostly linked to the activity observed. Nodes with corresponding hits in the GNPS MS/MS spectra library (with cosine > 0.65) are surrounded by a red circle, nodes which correspond to compounds isolated in the present study by a blue circle.

On the group of extracts corresponding to the genus *Coniochaeta*, the search script for compounds most linked to the observed activity (at 100 µg/mL in 48 h) brought out several ions, with FiBiCo scores greater than 2 (Figure 3). One of them (*m/z* 269.0448 at 12.8 min) returned a hit from the GNPS database as being emodin. In accordance with our results, emodin was previously reported to inhibit *Candida* biofilm [24]. Indeed, Janeczko et al. studied the activity of emodin against 50 strains of *C. albicans* and demonstrated that emodin was able to both inhibit early phase in the biofilm formation (adhesion) of 60% of the tested strains as well as inhibit already formed biofilms of 30% of these strains. Interestingly, another anthraquinone pointed out in the network, identified as endocrocin, was not emphasized by the FiBiCo script as an active compound. This compound has additional carboxylic function compared to emodin [25]. The node with a *m/z* 541.1126 at 12.7 min corresponded to another anthraquinone derivative, rugulosin, a dimeric anthraquinone.

For certain nodes highlighted by the FiBiCo script, no other molecule could be annotated whether using GNPS or ISDB databases. By inspecting MS spectra and retention times, three of these high-score ions could be linked to a same molecule (*m/z* 700.6101 as [M + FA-H]^−^, *m/z* 654.6044 as [M-H]^−^ and *m/z* 768.5977 as [M + TFA-H]^−^ at 20.9 min). The corresponding molecular formula C_40_H_81_NO_5_ suggested a ceramide-like compound. For the remaining interesting node, *m/z* 293.2116 at 14.1 min, it was found to match with the molecular formula C_18_H_32_O_4_ as [M-H_2_O-H]^−^, putatively annotated as oxygenated fatty acids. 

#### 3.4.2. Investigation of Extracts from *Biscognauxia* Genus

**Figure 4:**
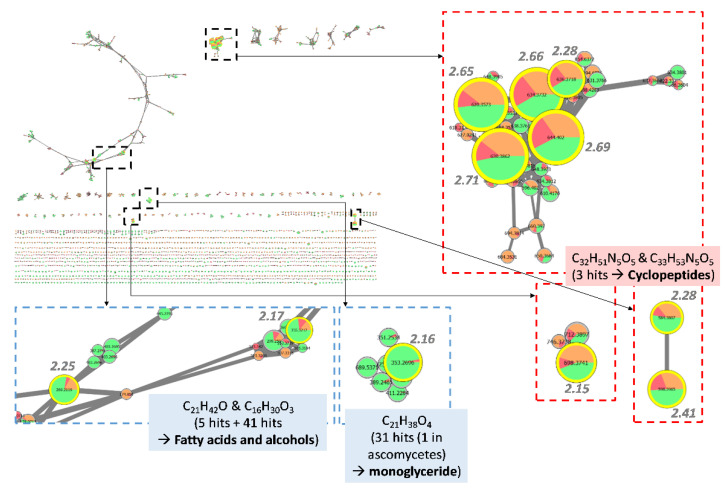
Feature-based molecular network corresponding to *Biscogniauxia* extracts. Nodes are filled with pie charts representing the mean peak areas for each feature in the 3 defined groups of activity (anti-maturation activity at 200 µg/mL in 48 h): low (red) for 0–30%, medium (orange) for 30–50% and high (green) for 50–100%. Node sizes are related to FiBiCo scores indicated in grey, highlighting compounds mostly linked to the activity observed.

The same approach was applied to *Biscogniauxia* extracts (Figure 4). In order to explore the anti-biofilm potential of *Biscognauxia* species, the threshold selected to consider an extract as active was raised to 200 µg/mL. In fact, even if less potent, extracts from this genus tended to show differences in activity with a dose-response profile, suggesting active metabolites might be present in very small quantities.

A cluster corresponding to several ions with a high score was highlighted. It appeared that this cluster with *m/z* 630.3862, 644.4020, 634.3732, 620.3573 together with two other clusters including *m/z* 598.3965, 584.3807 and 698.3741 actually corresponded to two compounds eluting at 13.5 and 13.8 min respectively. No hit was found by comparison to MS/MS spectra either in the GNPS nor the ISDB databases. Investigation of MS data suggested they were two cyclopeptides with their monoisotopic masses being 585.3880 and 599.4038, corresponding to the following ions: *m/z* 630.3862 and *m/z* 644.4020 for [M + FA-H]^−^, *m/z* 620.3573 and *m/z* 634.3732 for [M + Cl]^−^, *m/z* 584.3807 and *m/z* 598.3965 for [M-H]^−^, *m/z* 698.3741 and 712.3897 for [M + TFA-H]^−^. Further inspection of MS/MS data allowed us to propose the molecular formulae C_32_H_51_N_5_O_5_ and C_33_H_53_N_5_O_5_ for the two compounds, respectively. In fact, several fragments suggested neutral losses of Leu or Ile (355.2707 → 242.1876 → 129.1033) and Phe (389.2551 → 242.1876). An additional loss of Val or Iva (228.1714 → 129.1033) could be detected in the MS/MS spectrum of the compound which mass was 585.3880. This suggested the replacement of a Leu (or Ile) by a Val (or Iva) in the latter compound compared to the one with a mass at 599.4038. While some compounds described in the literature could match to these compounds such as the cyclo-(L-Phe-L-Leu1-L-Leu2-L-Leu-L-Ile) or the gameXpeptides A and B [26,27,28] the comparison of MS/MS fragmentation patterns did not completely correspond to these known compounds and further investigation would be needed to identify them. The other nodes highlighted such as the 353.2696 as [M-H]^−^at 15.6 min, 355.3217 as [M + FA-H]^−^ at 17.5 min and 269.2119 as [M-H]^−^ at 14.9 min corresponded to the molecular formulae C_21_H_38_O_4_, C_21_H_42_O and C_16_H_30_O_3_, respectively. The first one gave 31 hits in the DNP and only one when refined to ascomycetes, corresponding to a monoglyceride. The two others were mainly matched with fatty acids and alcohols.

#### 3.4.3. Investigation of Extracts from *Preussia* Genus

**Figure 5:**
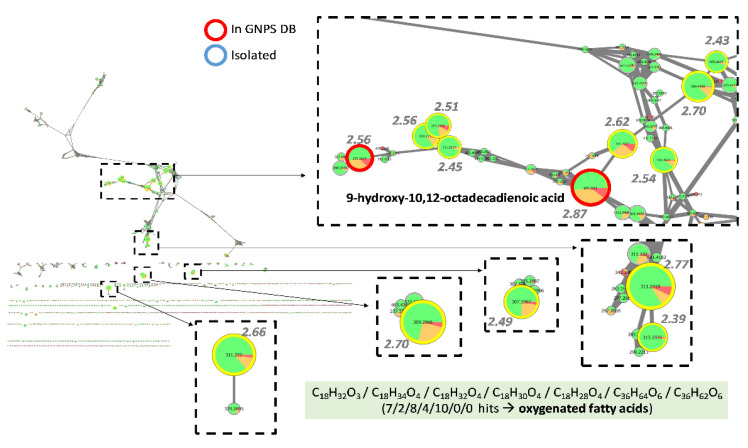
Feature-based molecular network corresponding to *Preussia* extracts. Nodes are filled with pie charts representing the mean peak areas for each feature in the 3 defined groups of activity (anti-maturation activity at 50 µg/mL in 48 h): low (red) for 0–40%, medium (orange) for 40–50% and high (green) for 50–100%. Node sizes are related to FiBiCo scores indicated in grey, highlighting compounds mostly linked to the activity observed.

*Preussia* extracts were particularly active against the biofilm studied and the threshold selected to consider the extract as active has been lowered at 50 µg/mL. Investigation of *Preussia* extracts mainly highlighted peaks corresponding to oxygenated fatty acids, which occurred to be massively produced in active extracts (Figure 5). GNPS database returned one hit as 9-hydroxy-10,12-octadecadienoic acid for the peak with the highest FiBiCo score (2.87), *m/z* 295.2271 at 13.69 min. Other closely related compounds with molecular formula matching with C_18_H_32_O_3_, C_18_H_34_O_4_, C_18_H_32_O_4_, C_18_H_30_O_4_ or C_18_H_28_O_4_ are also derivatives from the common fatty acid linoleic acid. Interestingly, we identified peaks such as *m/z* 589.4468 at 16.15 min and *m/z* 591.4622 at 16.52 min (with scores of 2.70 and 2.54 respectively), which did not match with any known compounds isolated from ascomycetes and which corresponded to potential dimers of the previously reported oxygenated fatty acids with C_36_H_64_O_6_ or C_36_H_62_O_6_ molecular formula. As they corresponded to the major ions on the MS1 spectra, clusters such as [2M-H_2_O-H]^−^ were excluded. Moreover, by closely inspecting MS1 spectra of these peaks, we could also detect adducts such as [M + Cl]^−^ at *m/z* 625.4240 or 627.4396 and [2M-H]^−^ clusters at *m/z* 1179.9001 and 1183.9353. Additionally, their MS^2^ fragmentation produced the monomer ions at 311.2227 and 313.2386

For other fungal genera, this approach could not be performed, due to the limited number of samples (<5) and/or to limited difference of activity between the samples, such as for *Anthostomella*, for which all 3 extracts were active.

#### 3.4.4. Dereplication of Other Active Extracts from *Anthostomella*

**Figure 6:**
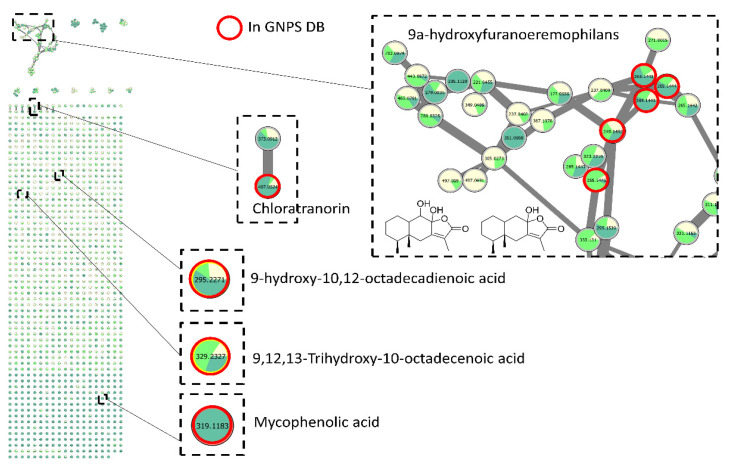
Feature-based molecular network corresponding to *Anthostomella* extracts. Nodes are filled with pie charts representing the mean peak areas for each feature in the different extracts investigated: MEA (light yellow), PDA (light green) and SAB (dark green).

For those extracts, molecular networking was still used for dereplication but without the scoring of the compounds by the FiBiCo script. In fact, few compounds could be annotated, revealing either the potential novelty potential for these species or the low number of MS/MS spectra of fungal molecules in the queried database (Figure 6). Indeed, the consistency of the database is still a critical point for dereplication [29]. Nevertheless, sesquiterpene lactone structures could be proposed for nodes corresponding to the biggest cluster of the molecular network, together with fatty acid derivatives.

Surprisingly, one node corresponding to *m/z* 407.0524 at 15.0 min was attributed to the classical lichenic compound chloratranorin. The related node with a *m/z* 373.0912 at 14.7 min could then be annotated as atranorin.

### 3.5. Isolation of Active Compounds

**Figure 7:**
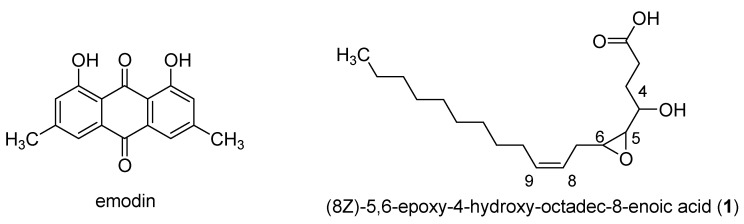
Chemical structures of compounds identified as actives by the FiBiCo script. Emodin was biosynthezised by *Coniochaeta* species and the fatty acid was isolated from *Preussia persica* extracts.

Following the results of the screening performed with the anti-biofilm tests, a large scale culture of *P. persica* has been carried out in MEA medium (10 L) in order to isolate fatty acids pointed out by the FiBiCo script tool. Purification of the extract (560 mg) by chromatographic silica gel column led to the isolation of one pure compound. This compound was determined to own the molecular formula C_18_H_32_O_4_ from the HRESIMS peak at *m/z* 311.2372 corresponding to three degrees of insaturation. The 1D NMR signals were attributed to one methyl (δ_C/H_ 14.4/0.89), eleven sp^3^ methylenes (δ_C/H_ 25.8–34.3/1.35), three oxygenated methines (δ_C/H_ 57.0/2.88, 60.2/2.75, 69.2), two olefinic methines (δ_C/H_ 125.9/5.51 and 134.9/5.51) and one carboxyl group (δ_C_ 174.8). These data indicated a fatty acid skeleton, and the straight chain was constructed following ^1^H-^1^H COSY correlations. HMBC correlations from H-2 (2.29) and H-3 (1.60) to C-1 (174.8) and ^1^H-^1^H COSY correlations from H-3 (1.60) to H-4 (3.44), H-4 to H-5 (2.75) and H-5 to H-6 (2.88) located the oxygenated functions in position C-4 (69.2), C-5 (60.2) and C-6 (57.0). The correlation of the methylene CH_2_-7 (2.37) with C-5 (60.2), C-6 (57.0), C-8 (125.9) and C-9 (132.9) further located the double bond between C-8 and C-9. Stereochemistry of the double bond has been assigned based on the coupling constant between H-8 and H-9. This constant value of 5.9 Hz corresponded to a *cis* position of the two protons (Supplementary material). A cis coupling was measured (4.1 Hz) between H-5 and H-6, the epoxide protons. A *threo* relationship was deduced for the hydroxyl and the epoxide proton at δ2.75 according to the coupling constant of 8.2 Hz between H-4 and H-5 [30]. The molecular formula together with ^13^C NMR chemical shifts clearly indicated the presence of an epoxy resulting from the oxidation of a double bond. This compound was thus elucidated as 8Z-5,6-epoxy-4-hydroxy-octadec-8-enoic acid (compound **1**, Figure 7) a poly-unsaturated fatty acid derived from linoleic acid. Production of oxygenated fatty acids is particularly favored in MEA media [31] with a factor 10 to 85 observed from SAB or PDA to MEA medium, on some selected features (Supplementary material Figures S20 and S21). MEA medium is rarely used for fungal cultures and allows a limited growth of fungi, as shown by the weak obtained quantities of extracts (Figure 1). The evaluation of the biological activity of this compound on biofilm showed an anti-biofilm activity lower than that of the extract (IC_50_ > 200 µM). To better assign the biological activity to a class of compounds, a second ethyl acetate extract of *P. persica* was prepared and submitted to a mixture composed of hexane, methanol and water resulting in the production of two extracts (E1 and E2). The evaluation of the anti-biofilm activity of these two extracts allowed us to attribute the activity to the more polar compounds present in extract E2 (inhibition percentage of 12% at 200 µg/mL for extract E1 and inhibition percentage of 50% at 50 µg/mL for extract E2 after 48 h of contact). The analysis of the non-polar compounds by GC-MS after derivatization showed the presence of middle chain fatty acids such as palmitic acid (C16:0), stearic acid (C18:0) and linoleic acid (C18:2) which are supposed to be non-responsible for the biological activity. The extract E2 mainly contained oxygenated derivatives of linoleic acid.

## 4. Discussion

The culture on three different media show that SAB medium is more suitable for the development of the fungal species and often led to high extraction rates of fungal metabolites. In contrast, the fungal growth is more difficult on MEA medium and led consequently to poor extraction rates. A second observation concerned the production of pigment metabolites that differed between media for a same species. This difference was particularly marked with *C. hoffmannii* and *P. varia*, whose pigment biosynthesis was increased on PDA medium (red for the first one and yellow for the second one). Among the 11 selected fungal species, the genera *Coniochaeta*, *Plectania* and *Preussia* afforded weak extraction rates. Also, only one species (*F. avenaceum*) has already been studied for its chemical content: cytotoxic metabolites have been isolated and characterized as eniatins, aurofusarin, chlamydosporol, fusarin C and moniliformin [32,33]. The chemical content of other species has not been studied yet.

The anti-biofilm activity of EtOAc extracts is better after 48 h of contact between the extract and the yeasts. Overall, a quarter of the tested extracts showed an activity on the maturation phase of the biofilm at 100 µg/mL. Only four extracts belonging to two different genera (*Preussia* and *Anthostomella*) were active on the mature biofilm at the same concentration. These results pointed out the difficulty to find out compounds active on a mature biofilm. The isolated clinical evaluation also showed a poor number of extracts without a strain-dependent activity.

Principal component analysis showed the consistency between chemical content of fungi cultivated in the three different media and taxonomy. The use of the OSMAC method to maximize the diversity of metabolites is particularly relevant for *Plectania* sp. Furthermore, by highlighting active extracts on the plot, it appeared that they did not obviously cluster together, suggesting that peaks (or features *m/z*_RT) responsible for the activity might be different from one genus to another. These observations then revealed the high potential of endolichenic fungi for their chemical diversity. This has been confirmed by the LC-MS^n^ analysis and the poor number of annotated peaks with the international MS databases.

The analysis of the chemical composition of the *Coniochaeta* genus show the presence of anthraquinoids compounds as well as ceramids, putatively implicated in the anti-biofilm activity. The production of anthraquinone derivatives (emodin and chrysophanol) has already been described for the endophytic *Coniochaeta velutina* cultivated on Potato Dextrose Broth (PDB) medium [34]. Anthraquinones such as emodin (Figure 7) have already been used for their anti-biofilm activity, but the cytotoxicity of such compounds could be a reason for further exploitation [35,36]. Ceramids are constituents of lipid rafts in filamentous fungi and are involved in growth, differentiation and response to environmental stress [37]. So far, only one type of fungal ceramide was found to have anti-biofilm activity [38]. Indeed, Vieira et al. showed that ceramide monohexosides inhibited the formation of biofilms made by methicillin-resistant Staphylococcus aureus (MRSA) bacteria. However, to our knowledge, no data yet exist that concern their activity on fungal biofilms. Thus, ceramids can easily interfere with the sphingolipid pathway and some similar compounds have be found as promising new anti-fungal drugs [39].

In the genus *Biscogniauxia*, metabolites such as azaphilones, polyketides and cyclopeptides have already been isolated [40,41]. Here, cyclopeptides have been highlighted by the FiBiCo script. Cyclopeptides have already been isolated from natural sources including ascomycetes fungi [42,43]. As previously mentioned, echinocandins which are hexacyclopeptides bearing lipophilic chain are already used for the treatment of fungal biofilms infections [44] and occurrence of such derivatives (pentacyclopeptides) in *Biscogniauxia* is definitely interesting. Nevertheless, cyclopeptides may be present in the extract at low concentration, not sufficient to endow the whole extract with an anti-biofilm activity. Their isolation followed by their biological evaluation would be of interest, but may need some optimization of the culture conditions.

In the genus *Preussia*, the most active extract, fatty acids are emphasized by the script. In a complete review on the chemotaxonomy of the genus *Preussia*, no fatty acids were reported [45]. This is the first report of the production of oxygenated fatty acids by the Genus *Preussia*. Kim et al. recently demonstrated that linoleic acid was able to reduce the formation of a *C. albicans* biofilm. However, these authors did not investigate its effect against preformed biofilms [46]. In perspective of this work, further investigations on these potential oxygenated fatty acid dimers will be considered. The anti-biofilm activity of such compounds has already been studied. Polyunsaturated fatty acids have been shown to induce the apoptosis in *C. albicans* and *C. dubliniensis* biofilms [47]. Also, bionectril B-D produced by *Bionectra ochroleuca* or myriocin isolated from *Myriococcum albomyces*, *Isaria sinclairii* and *Mycelia sterilia* have already been identified to prevent the formation of biofilms [48]. Furthermore, Nigam et al. showed the effect of 3R-hydroxy-tetradecaenoic acid on quorum sensing of *C. albicans* [33]. Thus, medium chain fatty-acids were recently shown to inhibit hyphal growth and cell aggregation on *C. albicans* biofilms. Their structural homology with farnesol interferes with the farnesol and sterol production in *C. albicans* lead to fungal dimorphism [49].

For the genus *Anthostomella*, only dereplication has been applied, which pointed to the presence of sesquiterpenes lactones and fatty acids. The presence of depsides is also noted. So far, sesquiterpene lactones have not been commonly described in endolichenic fungi, and anti-biofilm activity is still poorly explored [48,50]. To our knowledge, there are no data on the anti-biofilm potential of chloratranorin. Pompilio et al. demonstrated the ability of atranorin to inhibit preformed biofilms of MRSA in a concentration dependent manner [51] whereas Girardot et al. have shown no activity against *Candida* biofilms for this compound [15]. This raises questions about the role of endolichenic fungi in the production of lichenic compounds, which are not the subject of the current manuscript, but which should be further investigated.

With these results, we showed that the combination of molecular networking with biochemometrics allowed us to highlight the most interesting features in the dataset, which need to be focused on for dereplication and isolation work. This method proves to be a powerful tool to target active compounds as the results obtained were confirmed by literature data (i.e., emodin). While interesting natural products were revealed by this method, we need to be careful on the interpretation of results obtained as only the negative mode was chosen for MS analyses. Nonetheless, the peaks picked in the final matrices used for statistical analysis counted 3468, 2779 and 1225 features for *Coniochaeta*, *Biscogniauxia* and *Preussia* extracts, respectively. These numbers show that, even in the negative mode, the peak picking performed accounted for notable diversity in the extracts. Moreover, only peaks with MS/MS spectra recorded were analyzed. This could hide compounds which were not fragmented during the analysis. To overcome this issue, MS1 data were also analyzed by the FiBiCo script, and the hit lists were compared to make sure that any significant ion was missing. In fact, the results presented here were consistent with MS1 data.

It is important to note that only groups of extracts with a sufficient number of samples were treated in this way to obtain relevant results. For each ion of interest, scatter charts were obtained, and the correlation highlighted by the biochemometric analysis was shown by a tendency curve and the associated correlation factor (Supplementary material Figures S1–S19). This approach then allowed us to point quite rapidly to the most interesting families of natural fungal compounds that should be focused on for their anti-biofilm activities.

## 5. Conclusions

This work confirmed the chemical and the biological potential of endolichenic fungi. The metabolomic network performed on 40 extracts issued from 11 fungal species revealed a low number of annotated nodes. Most of the species selected have not yet been chemically studied, but the feature-based molecular network pointed out some interesting and biologically active molecular groups (like ceramids and cyclopeptides). Thus, further research on endolichenic metabolites is needed to expand the available MS-MS database, and to evaluate their biological activities. Among the endolichenic species studied herein, the anti-biofilm potential of oxygenated middle chain fatty acids is clearly highlighted as key fungal metabolites. Further studies on these classes of compound could be useful in order to established structure-activity relationships (degrees of oxygenation, degree of insaturation, size of the carbon chain) and/or to investigate the possible synergistic effect of such compounds.

## Figures and Tables

**Table 1:** Anti-maturation activity of endolichenic fungal extracts at 100 µg/mL on biofilm of *C. albicans* ATCC 28367. Extracts were added to yeast after the adhesion phase. The contact duration between biofilm and extracts was 24 h or 48 h.

Extract References	Fungi	Percent Inhibition Observed at 100 µg/mL	
		24 h	48 h
AP_PDA	*Anthostomella pinea*	47.8	64.2 *
AP_SAB	*Anthostomella pinea*	39.6	65.3 *
AP_MEA	*Anthostomella pinea*	4.7	71.3 *
BM_1_PDA	*Biscogniauxia mediterranea* (G1)	17.0	46.2
BM_1_SAB	*Biscogniauxia mediterranea* (G1)	37.0	30.7
BM_1_MEA	*Biscogniauxia mediterranea* (G1)	25.3	30.6
BM_2_PDA	*Biscogniauxia mediterranea* (G2)	0	25.5
BM_2_SAB	*Biscogniauxia mediterranea* (G2)	14.1	16.9
BN_1_PDA	*Biscogniauxia nummularia* (G1)	0	38.5
BN_1_SAB	*Biscogniauxia nummularia* (G1)	1.8	18.1
BN_1_MEA	*Biscogniauxia nummularia* (G1)	11.8	40.7
BN_2_PDA	*Biscogniauxia nummularia* (G2)	17.0	28.3
BN_2_SAB	*Biscogniauxia nummularia* (G2)	10.2	25.5
CD_PDA	*Coniochaeta decumbens*	10.5	39.6
CD_SAB	*Coniochaeta decumbens*	14.2	11.1
CD_MEA	*Coniochaeta decumbens*	38.7	65.8 *
CH_PDA	*Coniochaeta hoffmanii*	64.2 *	45.9
CH_SAB	*Coniochaeta hoffmanii*	0	18.4
CH_MEA	*Coniochaeta hoffmanii*	40.3	40.3
CL_PDA	*Coniochaeta lignicola*	32.2	64.6 *
CL_SAB	*Coniochaeta lignicola*	26.3	46.9
CL_MEA	*Coniochaeta lignicola*	19.7	48.5
FA_PDA	*Fusarium avenaceum*	0	19.5
FA_SAB	*Fusarium avenaceum*	8.3	28.2
FA_MEA	*Fusarium avenaceum*	22.0	29.5
PV_PDA	*Peziza varia*	24.6	40.5
PV_SAB	*Peziza varia*	29.8	35.8
PV_MEA	*Peziza varia*	48.7	44.8
PSP_MEA	*Plectania sp.*	48.7	59.4 *
PP_1_PDA	*Preussia persica* (G1)	37.4	55.0 *
PP_1_SAB	*Preussia persica* (G1)	32.5	37.8
PP_1_MEA	*Preussia persica* (G1)	25.7	46.0
PP_2_PDA	*Preussia persica* (G2)	25.6	50.6 *
PP_2_SAB	*Preussia persica* (G2)	28.1	54.4 *
PP_2_MEA	*Preussia persica* (G2)	62.1 *	64.4 *
SF_PDA	*Sordaria fimicola*	16.5	33.1
SF_SAB	*Sordaria fimicola*	33.7	23.0
SF_MEA	*Sordaria fimicola*	35.8	35.4

* Significantly different from the non-treated control (Mann-Whitney test *p* ≤ 0.0002).

**Table 2:** Anti-biofilm activity of endolichenic fungal extracts at 100 µg/mL on biofilm of *C. albicans* ATCC 28367. Extracts were added on a 24 h-old biofilm. The contact duration between biofilm and extracts was 24 h or 48 h.

Extract References	Fungi	Percent Inhibition Observed at 100 µg/mL	
		24 h	48 h
AP_PDA	*Anthostomella pinea*	64.0 *	50.7 *
AP_SAB	*Anthostomella pinea*	8.8	59.9 *
AP_MEA	*Anthostomella pinea*	60.0 *	68.8 *
CD_MEA	*Coniochaeta decumbens*	17.4	35.9
CL_PDA	*Coniochaeta lignicola*	30.4	46.2
PSP_MEA	*Plectania* sp.	0	25.8
PP_1_PDA	*Preussia persica* (G1)	0	0
PP_2_PDA	*Preussia persica* (G2)	0	0
PP_2_SAB	*Preussia persica* (G2)	0	0
PP_2_MEA	*Preussia persica* (G2)	63.3 *	67.9 *

* Significantly different from the non-treated control (Mann-Whitney test *p* < 0.0001).

**Table 3:** Anti-biofilm activity of endolichenic fungal extracts at 100 µg/mL on biofilms formed by clinical isolates (CI) of *C. albicans*. Extracts were added to 24 h-old biofilms. The contact duration between extracts and biofilms was 48 h.

Extract References	CI1	CI2	CI3
	48 h	48 h	48 h
AP_PDA	58.6 *	50.3 *	29.6
AP_SAB	52.2 *	5.0	8.2
AP_MEA	59.5 *	40.5	19.0
PP_2_MEA	67.9 *	75.3 *	56.9 *

* Significantly different from the non-treated control (Mann-Whitney test *p* < 0.0001).

## Data Availability

Not applicable.

## Supplementary Materials

The following supporting information can be downloaded at: https://www.mdpi.com/article/10.3390/jof8101012/s1, Figure S1: scatter plot presenting peak areas of feature *m/z* 269.0448 in *Coniochaeta* samples; Figure S2: scatter plot presenting peak areas of features *m/z* 654.6044, 700.6101 and 768.5977 in *Coniochaeta* samples; Figure S3: scatter plot presenting peak areas of feature *m/z* 298.02116 in *Coniochaeta* samples; Figure S4: scatter plot presenting peak areas of feature *m/z* 541.1126 in *Coniochaeta* samples Figure S5: scatter plot presenting peak areas of features *m/z* 584.3807, 620.3573, 630.3862 in *Biscogniauxia* samples; Figure S6: scatter plot presenting peak areas of features *m/z* 598.3965, 634.3732, 644.4020 in *Biscogniauxia* samples; Figure S7: scatter plot presenting peak areas of feature *m/z* 269.2119 in *Biscogniauxia* samples; Figure S8: scatter plot presenting peak areas of feature *m/z* 295.2271 in *Preussia* samples; Figure S9: scatter plot presenting peak areas of feature *m/z* 311.2219 in *Preussia* samples; Figure S10: scatter plot presenting peak areas of feature *m/z* 589.4468 in *Preussia* samples; Figure S11: scatter plot presenting peak areas of feature *m/z* 309.2066 in *Preussia* samples; Figure S12: scatter plot presenting peak areas of feature *m/z* 311.222 in *Preussia* samples; Figure S13: scatter plot presenting peak areas of feature *m/z* 311.2222 in *Preussia* samples; Figure S14: scatter plot presenting peak areas of feature *m/z* 295.2274 in *Preussia* samples; Figure S15: scatter plot presenting peak areas of feature *m/z* 293.2111 in *Preussia* samples; Figure S16: scatter plot presenting peak areas of feature *m/z* 591.4622 in *Preussia* samples; Figure S17: scatter plot presenting peak areas of feature *m/z* 298.2115 in *Preussia* samples; Figure S18: scatter plot presenting peak areas of feature *m/z* 307.1907 in *Preussia* samples; Figure S19: scatter plot presenting peak areas of feature *m/z* 311.2217 in *Preussia* samples; Figure S20: extracted ion chromatogram for *m/z* 311.2219; Figure S21: peak area of the three major peaks for *m/z* 311.2220 in the different extracts of *Preussia* species; Figure S22: ^1^H NMR spectrum for compound 8Z-5,6-epoxy-4-hydroxy-octadec-8-enoic acid; Figure S23 and S24: ^13^ C NMR spectra for compound 8Z-5,6-epoxy-4-hydroxy-octadec-8-enoic acid; Figure S25 and S26: 2D NMR COSY spectra for compound 8Z-5,6-epoxy-4-hydroxy-octadec-8-enoic acid; Figure S27 and S28: 2D NMR HSQC spectra for compound 8Z-5,6-epoxy-4-hydroxy-octadec-8-enoic acid; Figure S29 and S30: 2D NMR HMBC spectra for compound 8Z-5,6-epoxy-4-hydroxy-octadec-8-enoic acid; Table S1: fungal strains description; Table S2: endolichenic fungi culture.
