# Supplementary material for: Access to Anti-Biofilm Compounds from Endolichenic Fungi Using a Bioguided Networking Screening
Source: J Fungi (Basel). 2022 Sep 27;8(10):1012. doi: 10.3390/jof8101012 (PMC9604612; doi:10.3390/jof8101012)

SUPPLEMENTARY INFORMATIONS

Access to anti-biofilm compounds from endolichenic fungi using a bioguided networking screening.

Seinde Toure[a], Marion Millot[a*], Lucie Ory[b], Catherine Roulier[b], Zineb Khaldi[a], Valentin Pichon[a], Marion Girardot[c], Christine Imbert[c], Lengo Mambu[a]

[a] Univ. Limoges, Laboratoire PEIRENE, UR 22722, F-87000 Limoges, France

[b] Nantes Université, Institut des Substances et Organismes de la Mer (ISOMer), UR 2160, F-44000, Nantes, France

[c] Univ. Poitiers, Laboratoire Ecologie et Biologie des Interactions (EBI), UMR CNRS 7267, F-86000, Poitiers, France

Table S1. Fungal strains description

Species	Class	Order	Family	Accession number	Lichen source
Anthostomella pinea	Sordariomycetes	Xylariales	Xylariaceae	MG543933	*Evernia prunastri*
Biscogniauxia nummularia	Sordariomycetes	Xylariales	Xylariaceae	MG543960	*Ramalina fastigiata*
Biscogniauxia nummularia	Sordariomycetes	Xylariales	Xylariaceae	MG543965	*Ramalina fastigiata*
Biscogniauxia mediterranea	Sordariomycetes	Xylariales	Xylariaceae	MG543959	*Ramalina fastigiata*
Biscogniauxia mediterranea	Sordariomycetes	Xylariales	Xylariaceae	MG543963	*Ramalina fastigiata*
Coniochaeta hoffmannii	Sordariomycetes	Coniochaetales	Coniochaetaceae	MG543962	*Ramalina fastigiata*
Coniochaeta lignicola	Sordariomycetes	Coniochaetales	Coniochaetaceae	MG543934	*Ramalina fastigiata*
Coniochaeta decumbens	Sordariomycetes	Coniochaetales	Coniochaetaceae	MG543958	*Ramalina fastigiata*
Fusarium avenaceum	Sordariomycetes	Hypocreales	Nectriaceae	MG543945	*Pleurosticta acetabulum*
Peziza varia	Pezizomycetes	Pezizales	Pezizaceae	MG917006	*Nephroma laevigatum*
Plectania melastoma	Pezizomycetes	Pezizales	Sarcosomataceae	MG543948	*Pleurosticta acetabulum*
Preussia persica	Dothideomycetes	Pleosporales	Sporormiaceae	MG543927	*Evernia prunastri*
Preussia persica	Dothideomycetes	Pleosporales	Sporormiaceae	MG543964	*Ramalina fastigiata*
Sordaria fimicola	Sordariomycetes	Sordariales	Sordariaceae	MG543930	*Evernia prunastri*

Table S2. Endolichenic fungi culture

Identification	Code	PDA	SAB	MEA	Comments
Anthostomella pinea	EP22	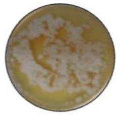		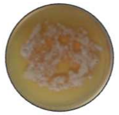	Growth very slow. Extraction after xx weeks
Biscogniauxia nummularia Genotype 1	RF09	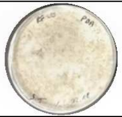	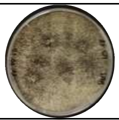	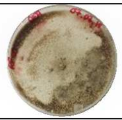	
Biscogniauxia nummularia Genotype 2	RF11	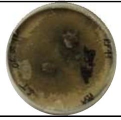	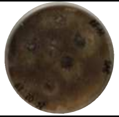		
Biscogniauxia mediterranea Genotype 1	RF13		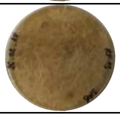		
Coniochaeta hoffmannii	RF20	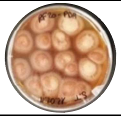	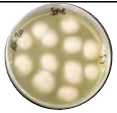	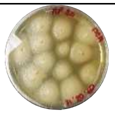	
Biscogniauxia mediterranea Genotype 2	RF26		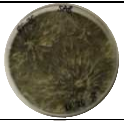		
Coniochaeta decumbens	RF31	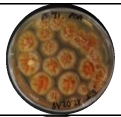	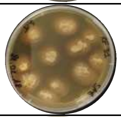		
Coniochaeta lignicola	EP25	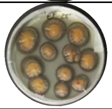	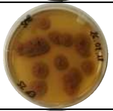	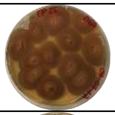	
Fusarium avenaceum	PA25	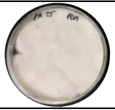	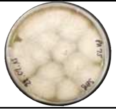	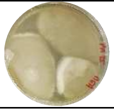	

Plectania melastoma	PA39	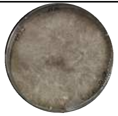	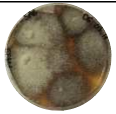	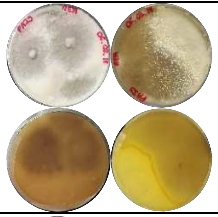	
Peziza varia	Gir02	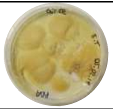	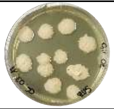	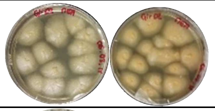	On MEA medium, color white-brown or rosish
Sordaria fimicola (1)	EP01	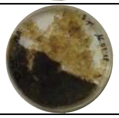	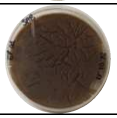	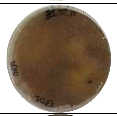	
Preussia persica (1)	RF28			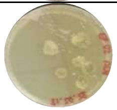	No growth from cryotubes
Preussia persica (2)	EP02	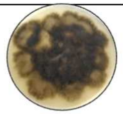		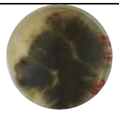	

Figure S1. Scatter plot presenting peak areas of highlighted feature *m/z* 269.0448 at 12.8 min (emodin) in correlation with the activity of the corresponding extracts of *Coniochaeta* samples. Pearson correlation coefficient of the linear regression r² is given.

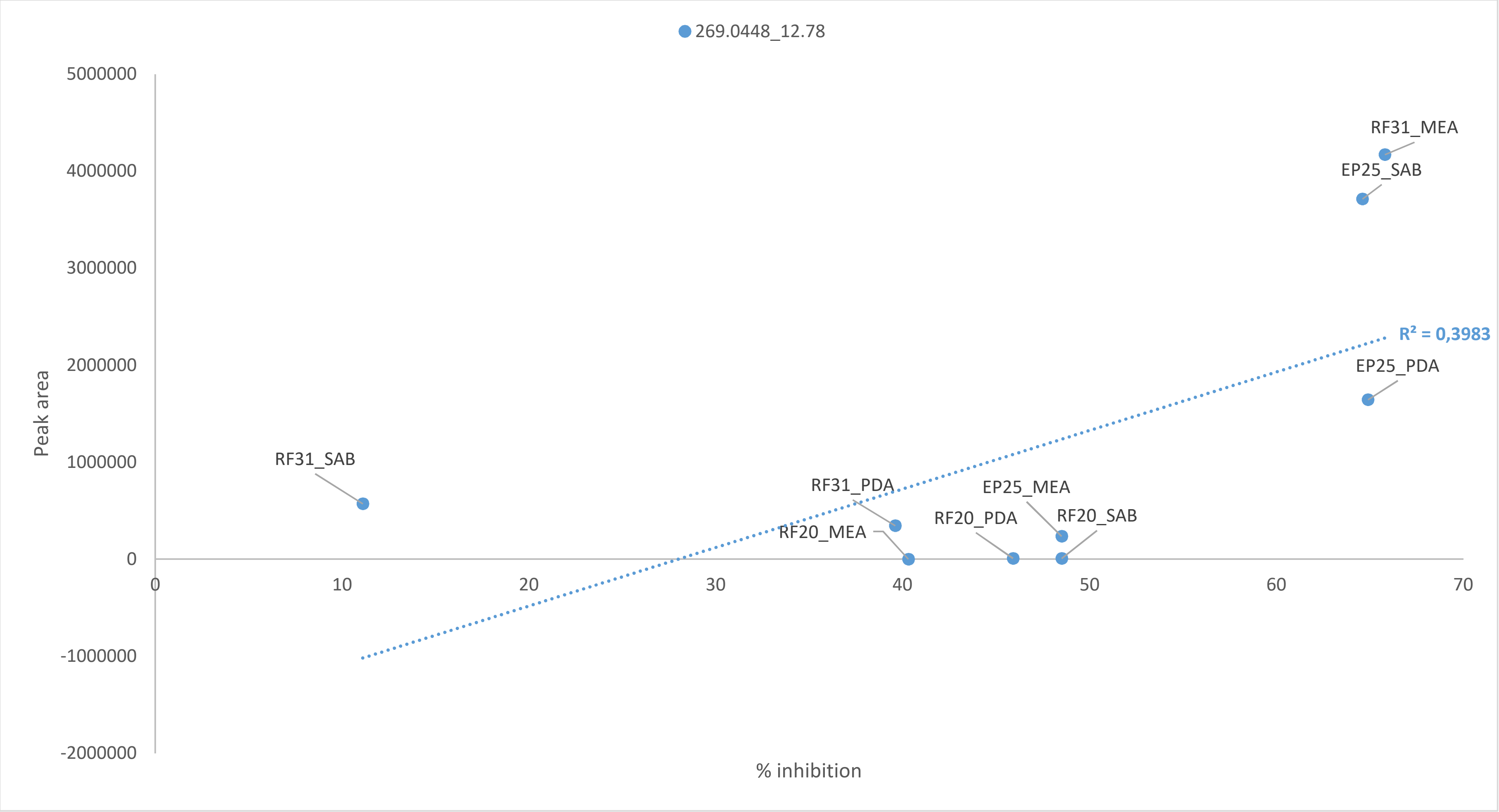

Figure S2. Scatter plot presenting peak areas of highlighted features *m/z* 654.6044, 700.6101 and 768.5977 at 21.0 min in correlation with the activity of the corresponding extracts of *Coniochaeta* samples. Pearson correlation coefficient of the linear regression r² is given.

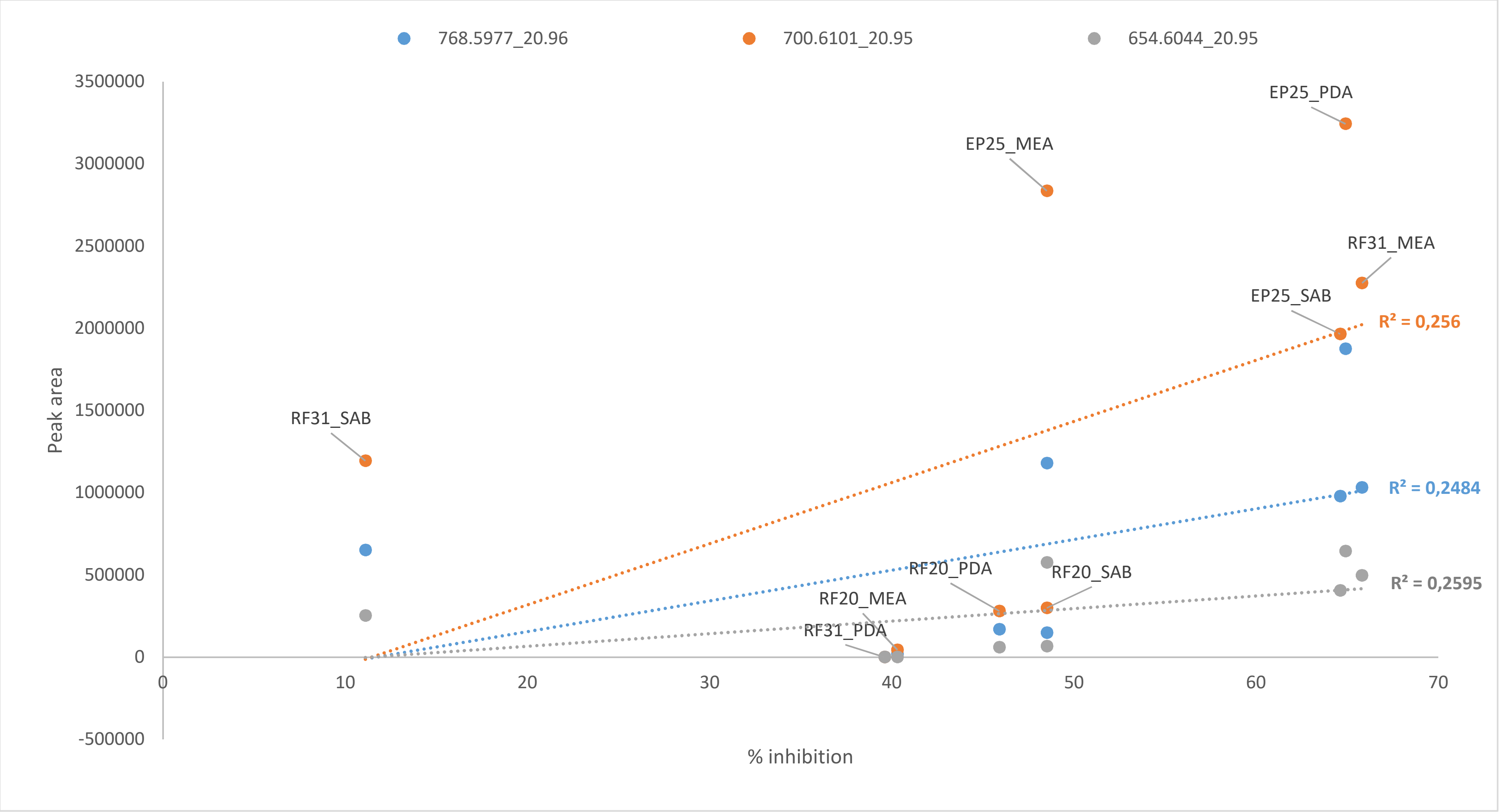

Figure S3. Scatter plot presenting peak areas of highlighted feature *m/z* 298.2116 at 14.1 min in correlation with the activity of the corresponding extracts of *Coniochaeta* samples. Pearson correlation coefficient of the linear regression r^2 is given.

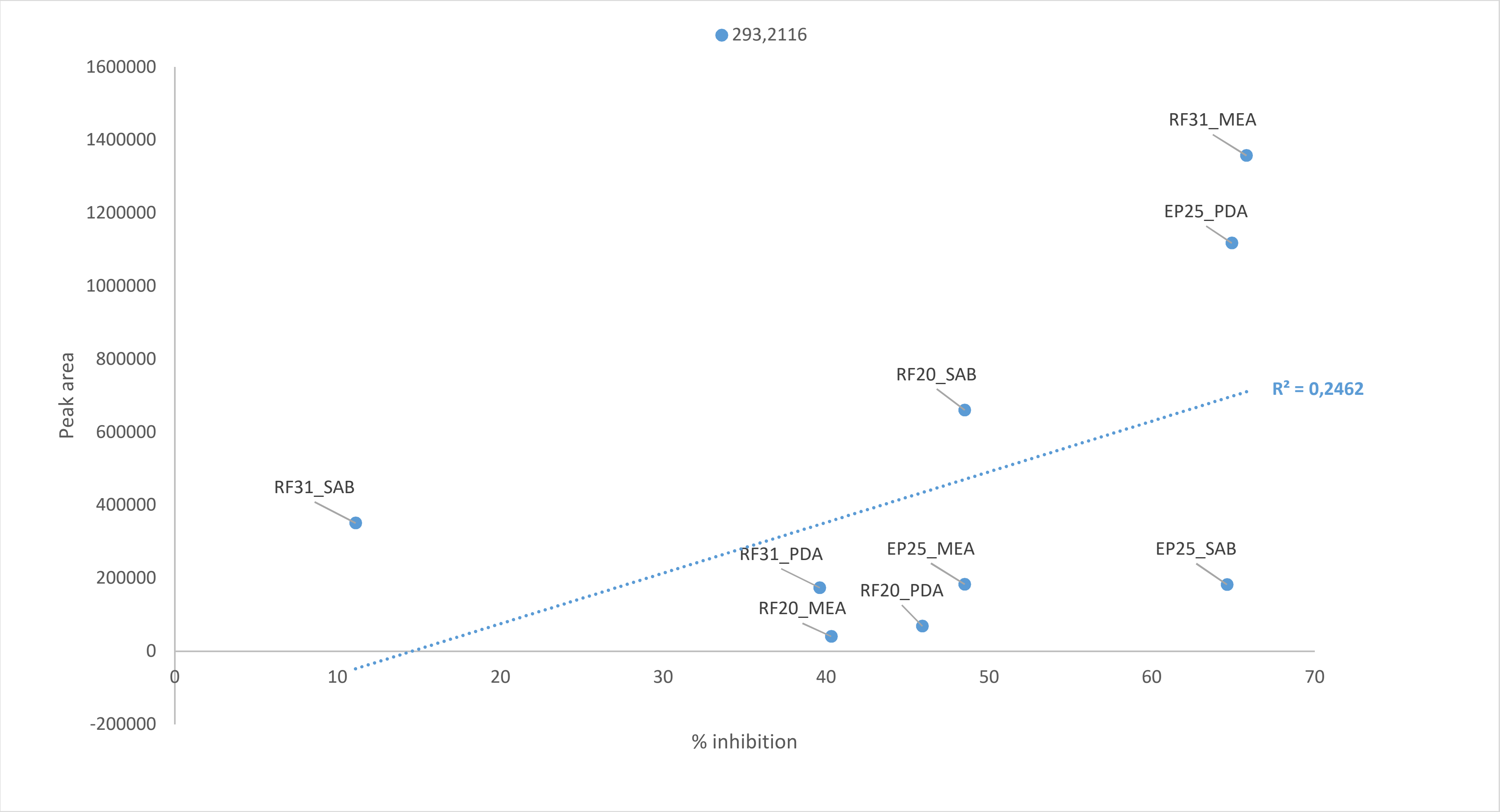

Figure S4. Scatter plot presenting peak areas of highlighted feature *m/z* 541.1126 at 12.7 min (emodin) in correlation with the activity of the corresponding extracts of *Coniochaeta* samples. Pearson correlation coefficient of the linear regression r² is given.

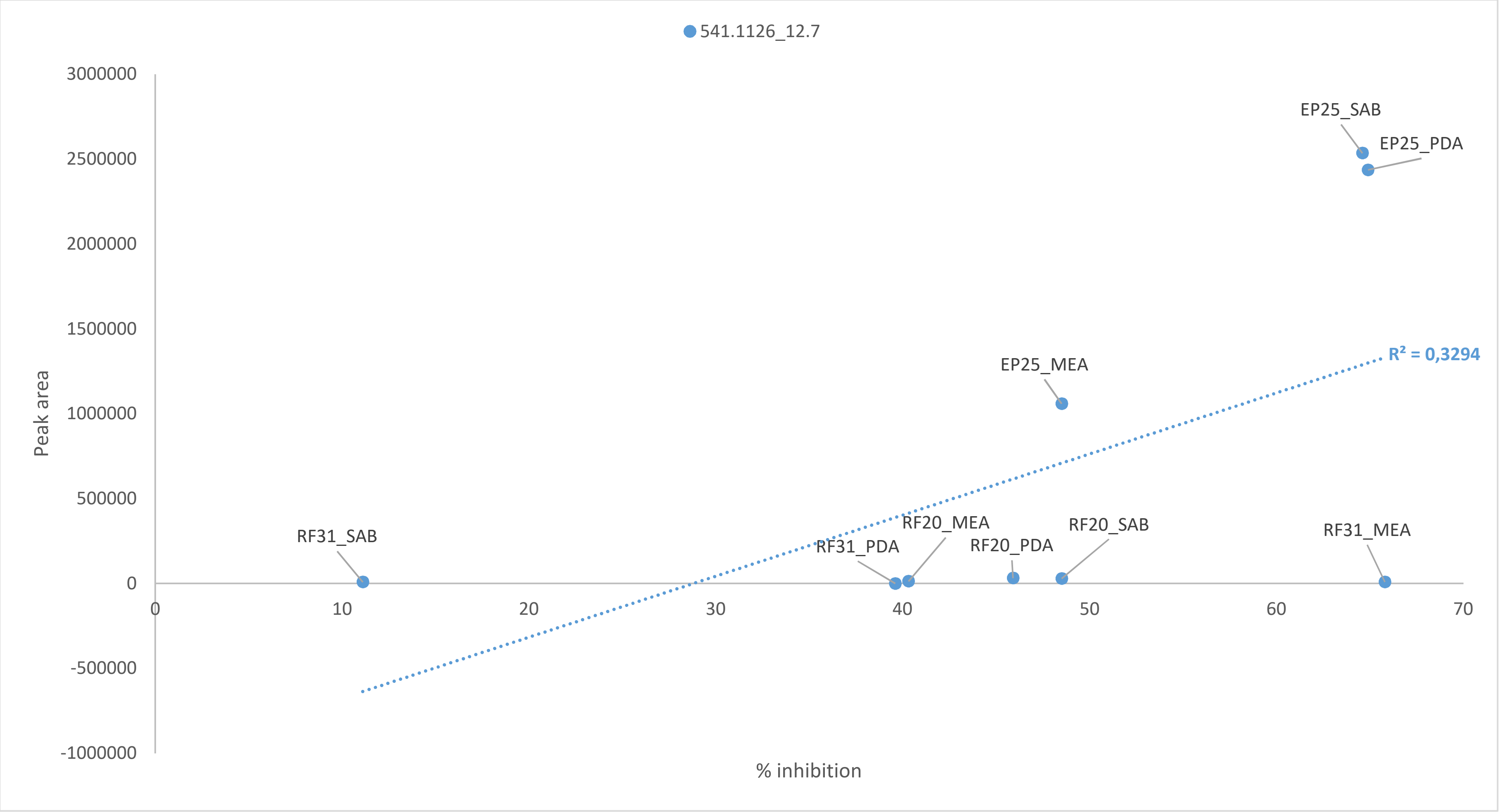

Figure S5. Scatter plot presenting peak areas of highlighted features *m/z* 584.3807, 620.3573 and 630.3862 at 13.5 min in correlation with the activity of the corresponding extracts of *Biscogniauxia* samples. Pearson correlation coefficient of the linear regression r^2 is given.

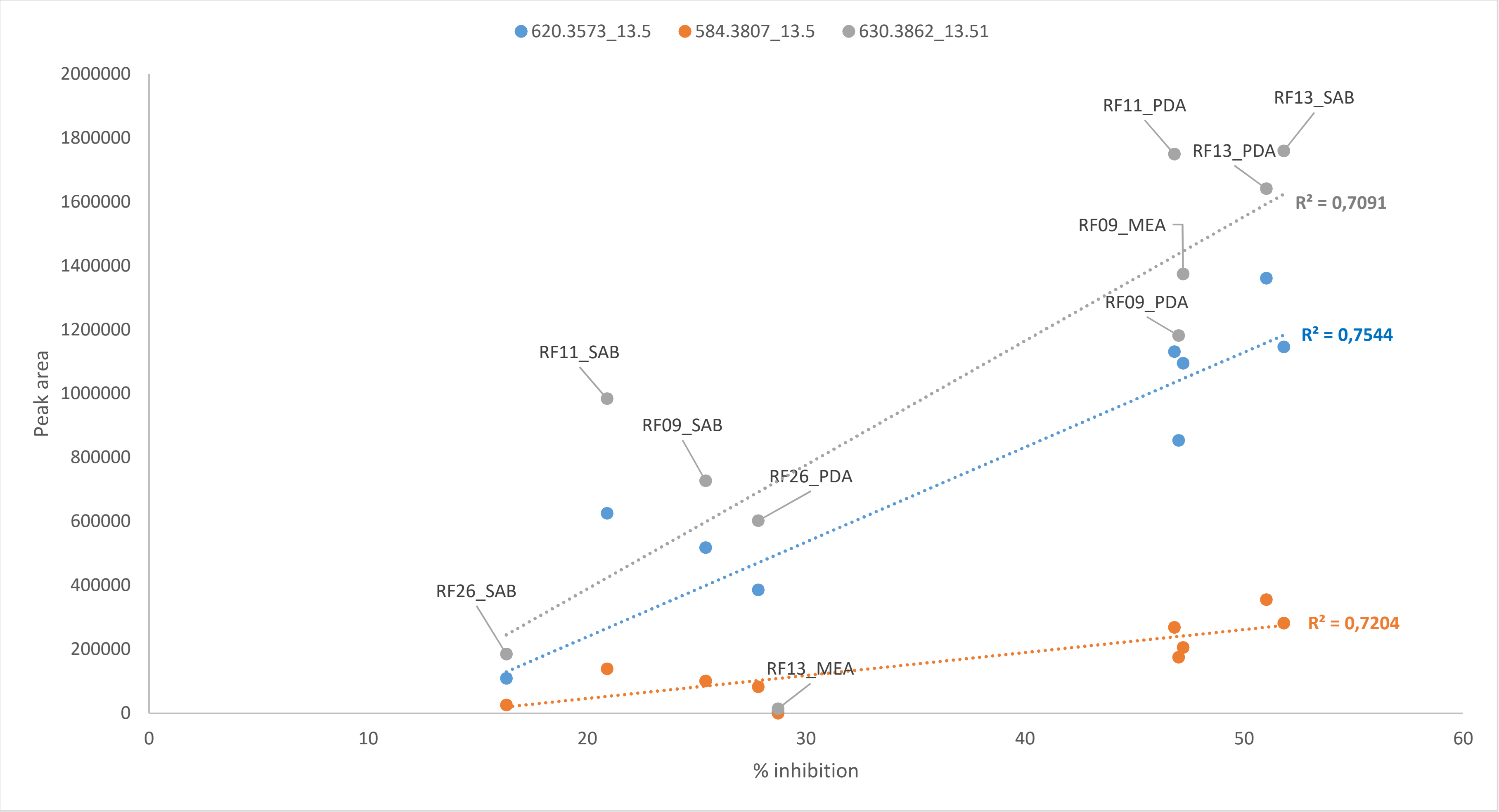

Figure S6. Scatter plot presenting peak areas of highlighted features *m/z* 598.3965, 634.3732 and 644.4020 at 13.8 min in correlation with the activity of the corresponding extracts of *Biscogniauxia* samples. Pearson correlation coefficient of the linear regression r² is given.

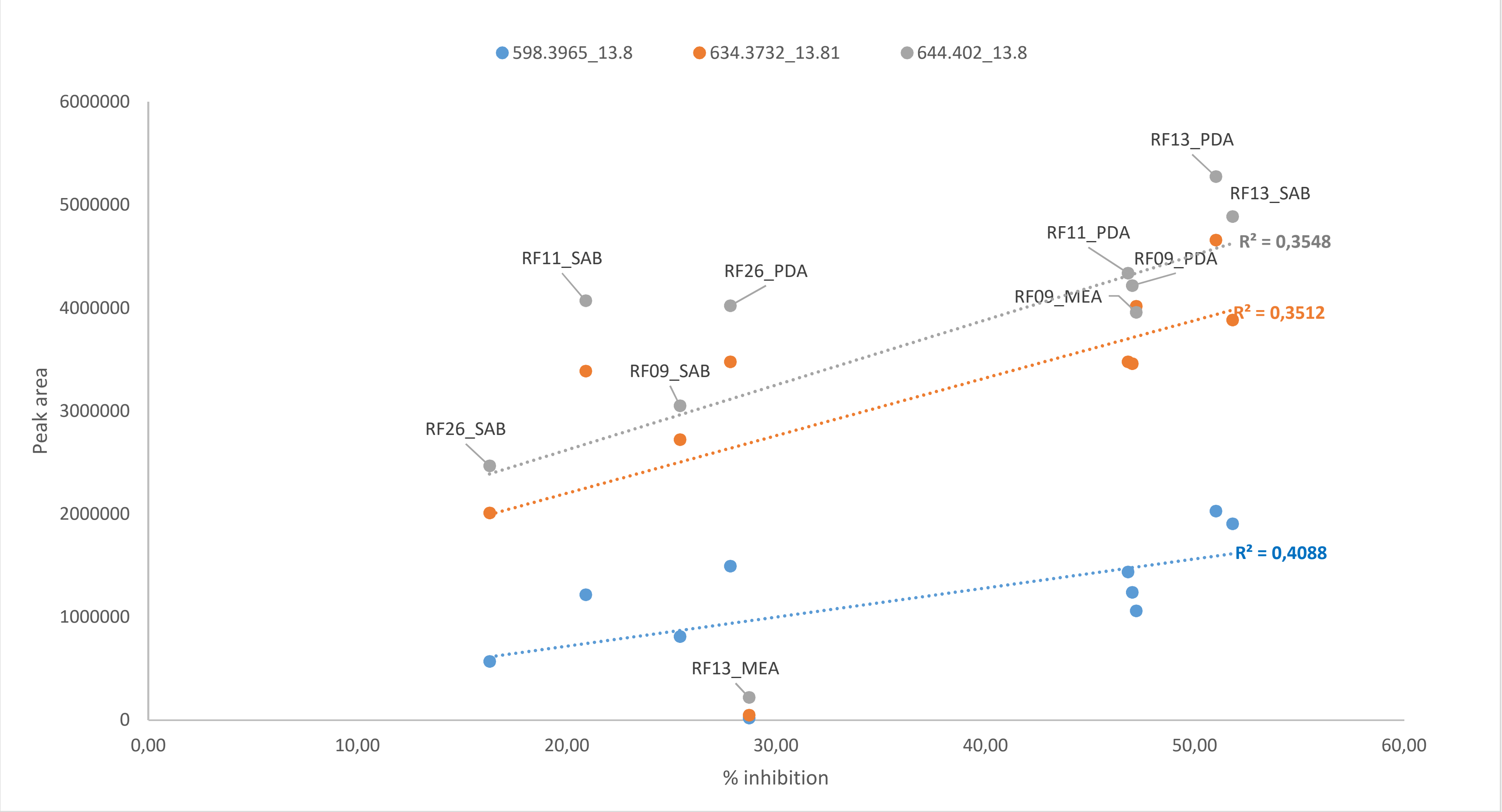

Figure S7. Scatter plot presenting peak areas of highlighted feature *m/z* 269.2119 at 14.9 min in correlation with the activity of the corresponding extracts of *Biscogniauxia* samples. Pearson correlation coefficient of the linear regression r² is given.

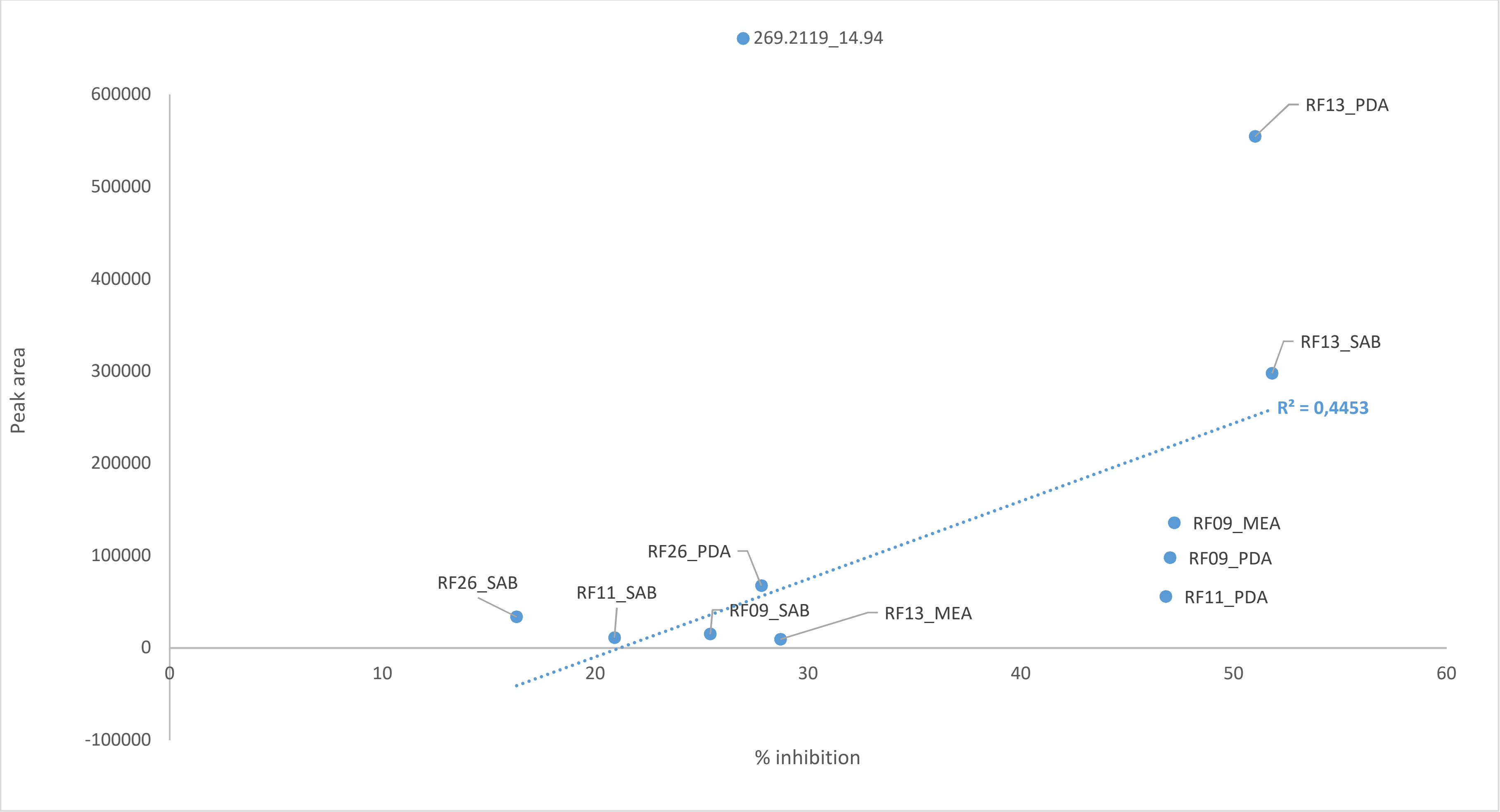

Figure S8. Scatter plot presenting peak areas of highlighted feature *m/z* 295.2271 at 13.7 min in correlation with the activity of the corresponding extracts of *Preussia* samples. Pearson correlation coefficient of the linear regression r² is given.

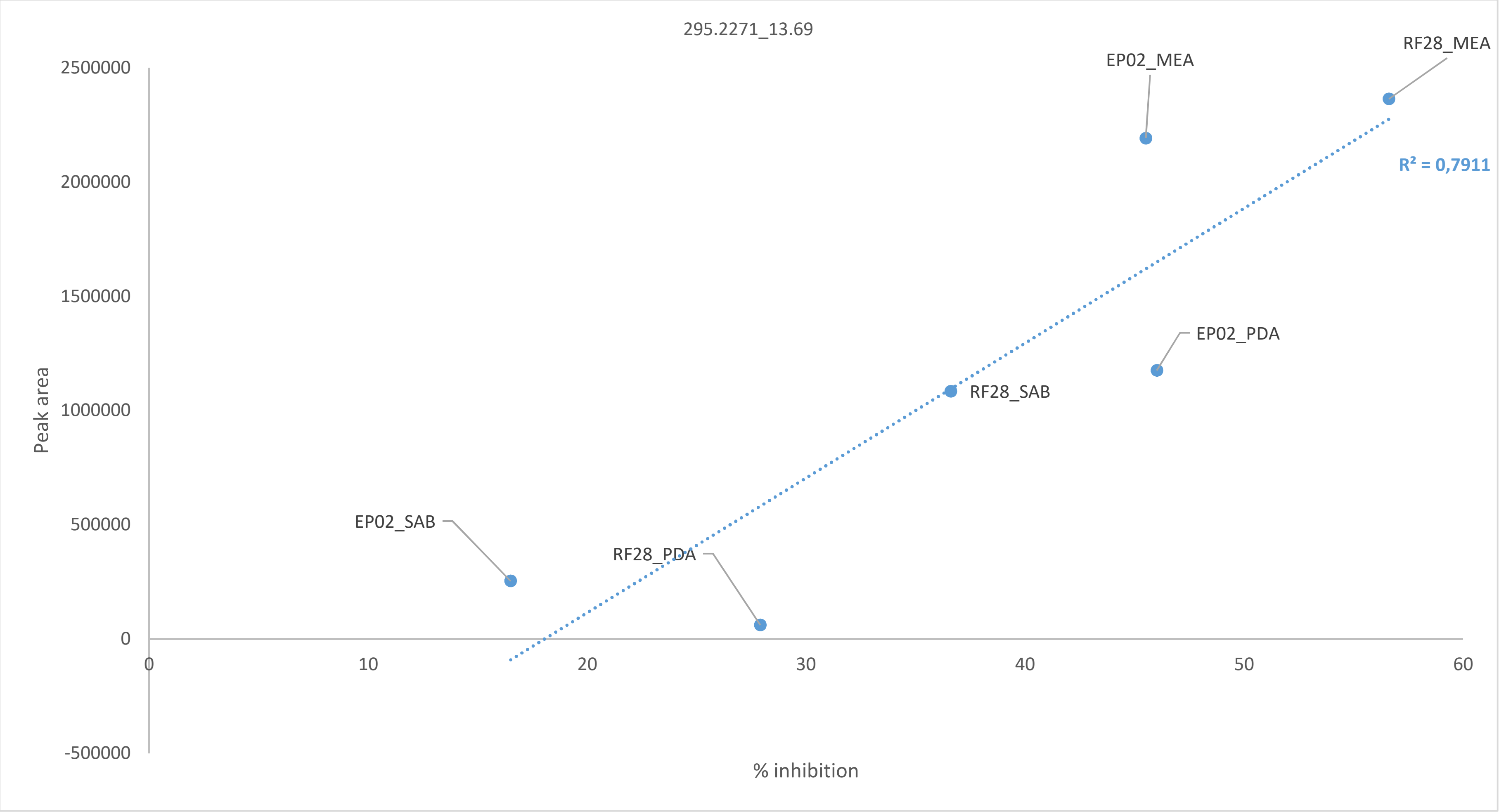

Figure S9. Scatter plot presenting peak areas of highlighted feature *m/z* 311.2219 at 13.0 min in correlation with the activity of the corresponding extracts of *Preussia* samples. Pearson correlation coefficient of the linear regression r^2 is given.

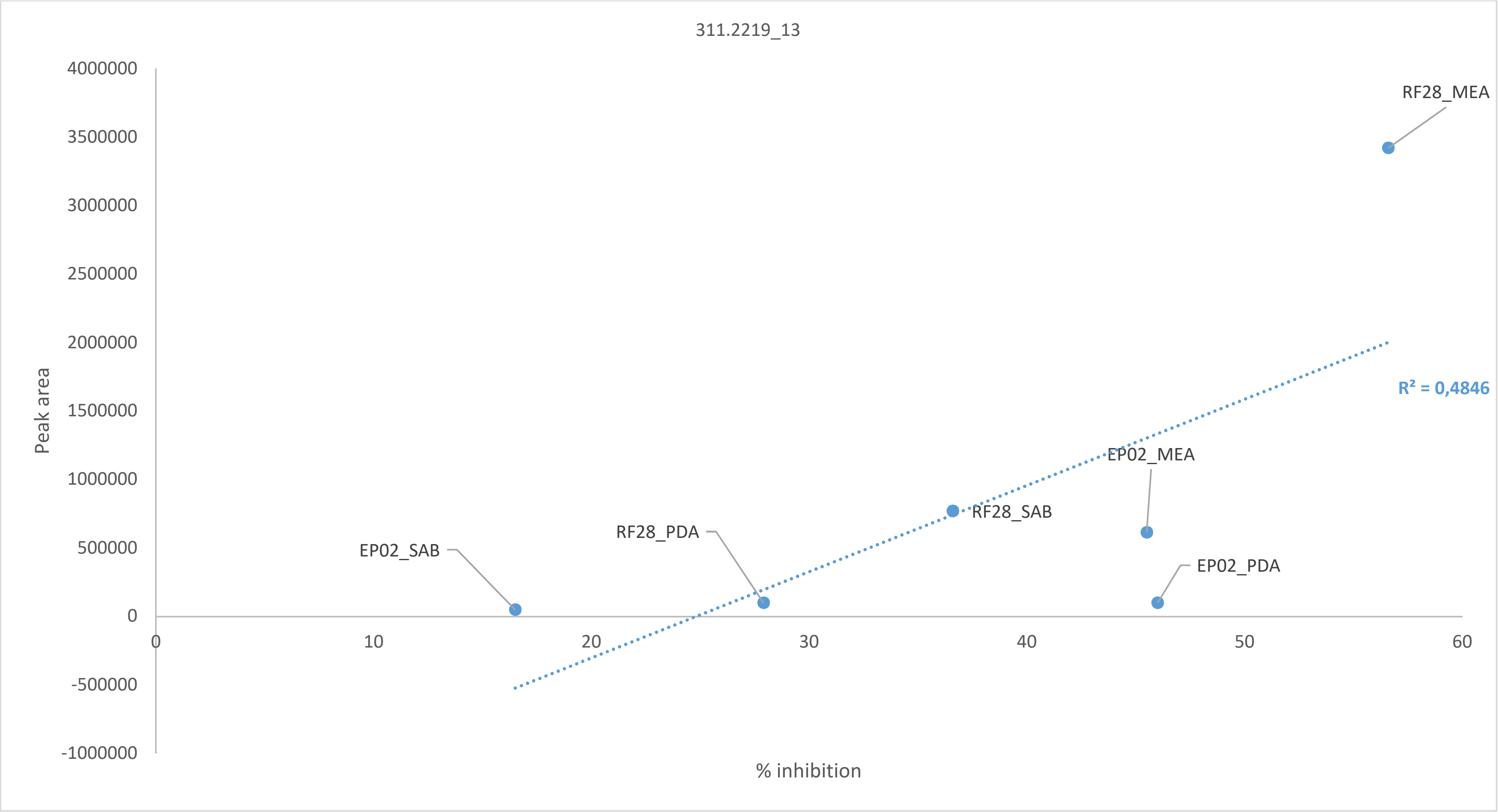

Figure S10. Scatter plot presenting peak areas of highlighted feature *m/z* 589.4468 at 16.2 min in correlation with the activity of the corresponding extracts of *Preussia* samples. Pearson correlation coefficient of the linear regression r² is given.

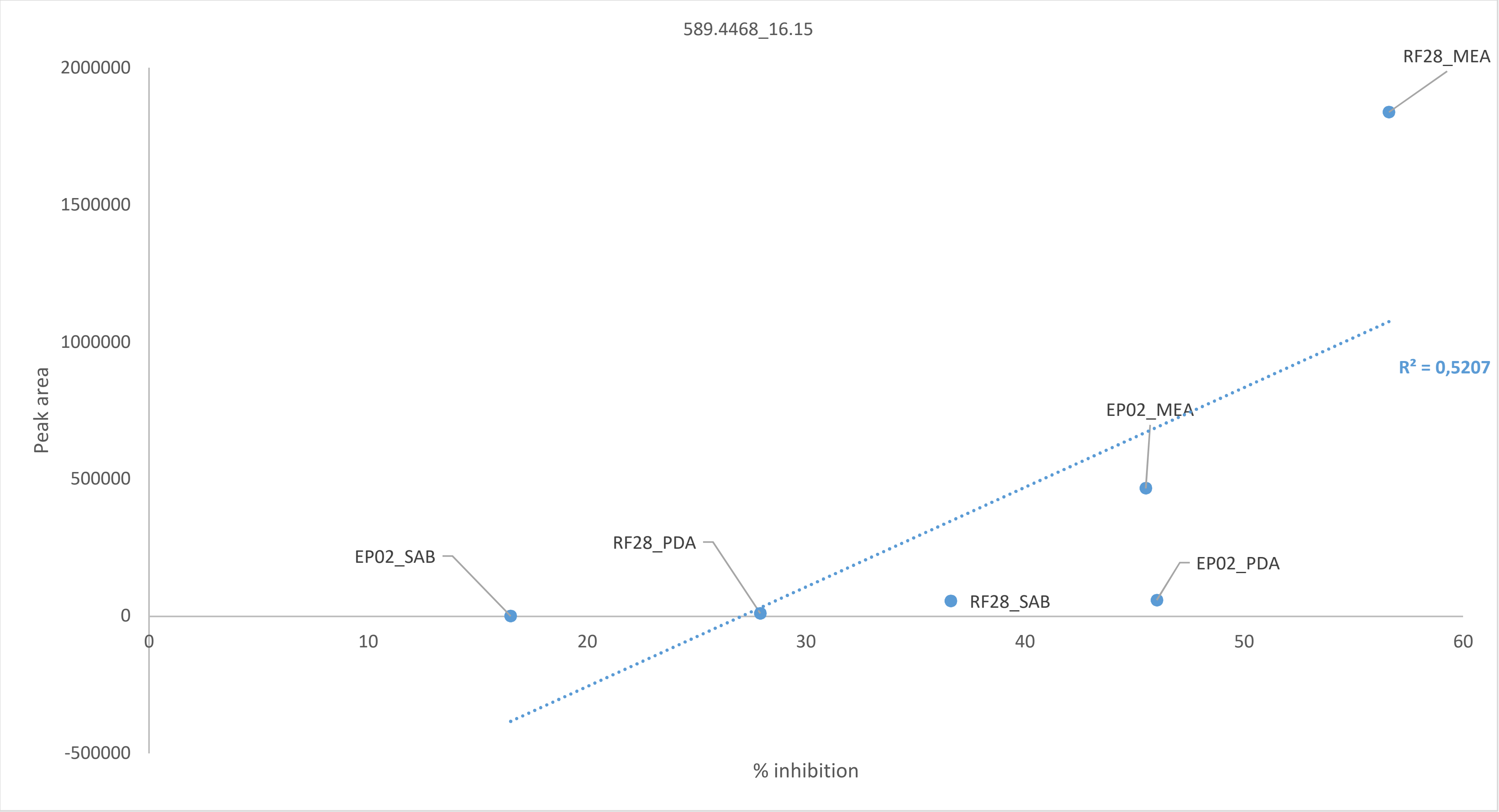

Figure S11. Scatter plot presenting peak areas of highlighted feature *m/z* 309.2066 at 12.1 min in correlation with the activity of the corresponding extracts of *Preussia* samples. Pearson correlation coefficient of the linear regression r² is given.

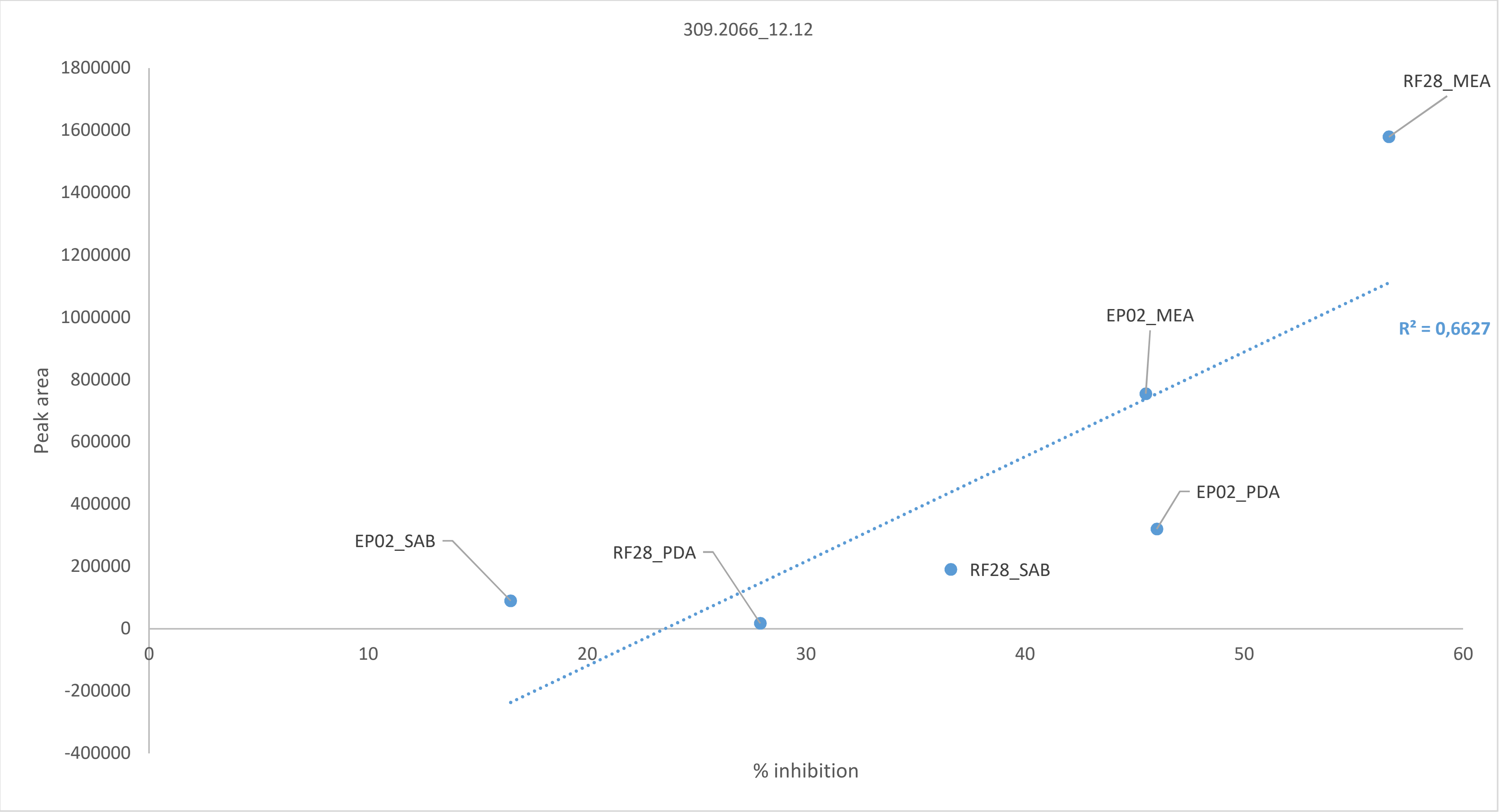

Figure S12. Scatter plot presenting peak areas of highlighted feature *m/z* 311.222 at 12.8 min in correlation with the activity of the corresponding extracts of *Preussia* samples. Pearson correlation coefficient of the linear regression r^2 is given.

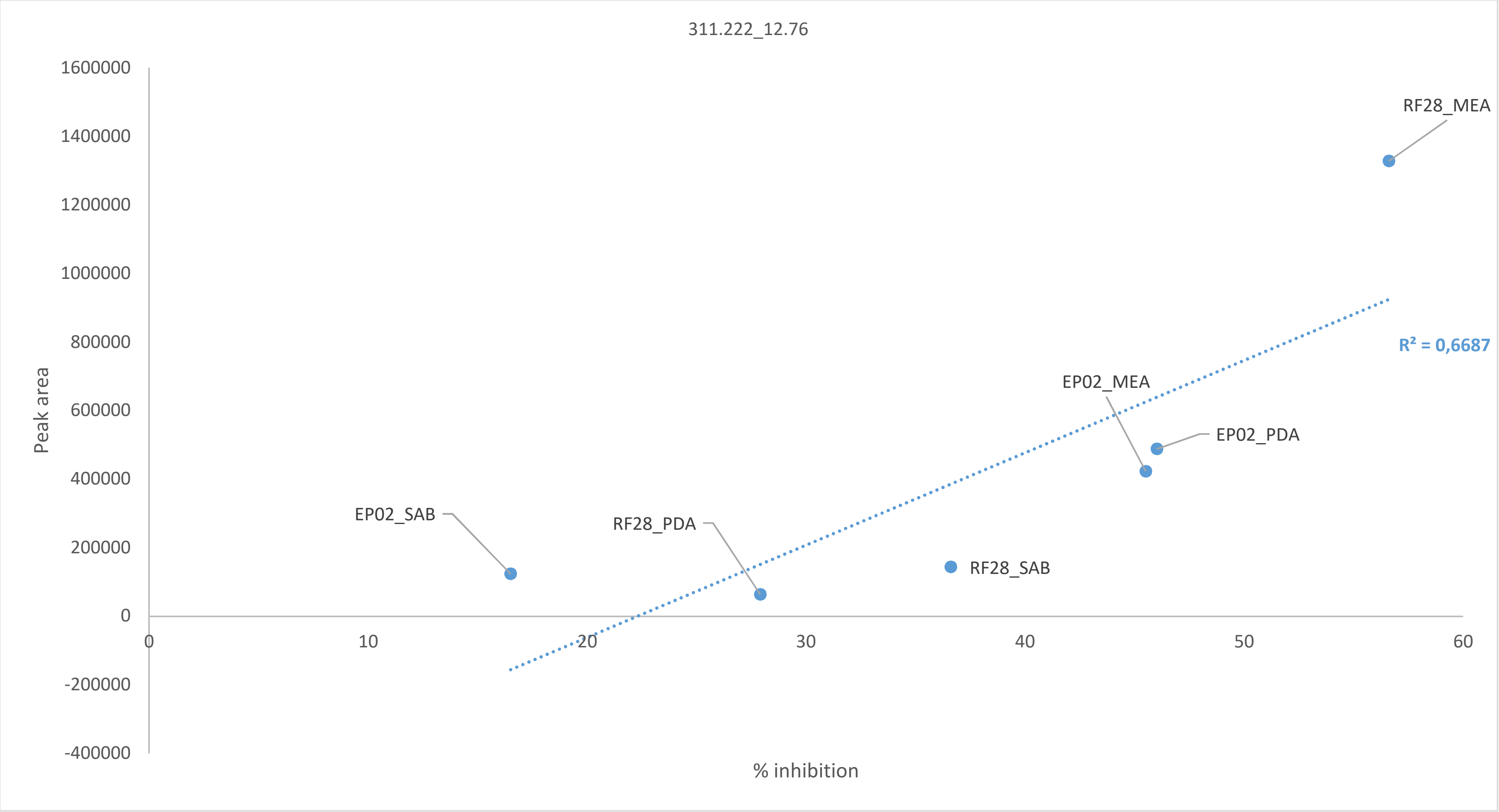

Figure S13. Scatter plot presenting peak areas of highlighted feature *m/z* 311.2222 at 12.0 min in correlation with the activity of the corresponding extracts of *Preussia* samples. Pearson correlation coefficient of the linear regression r² is given.

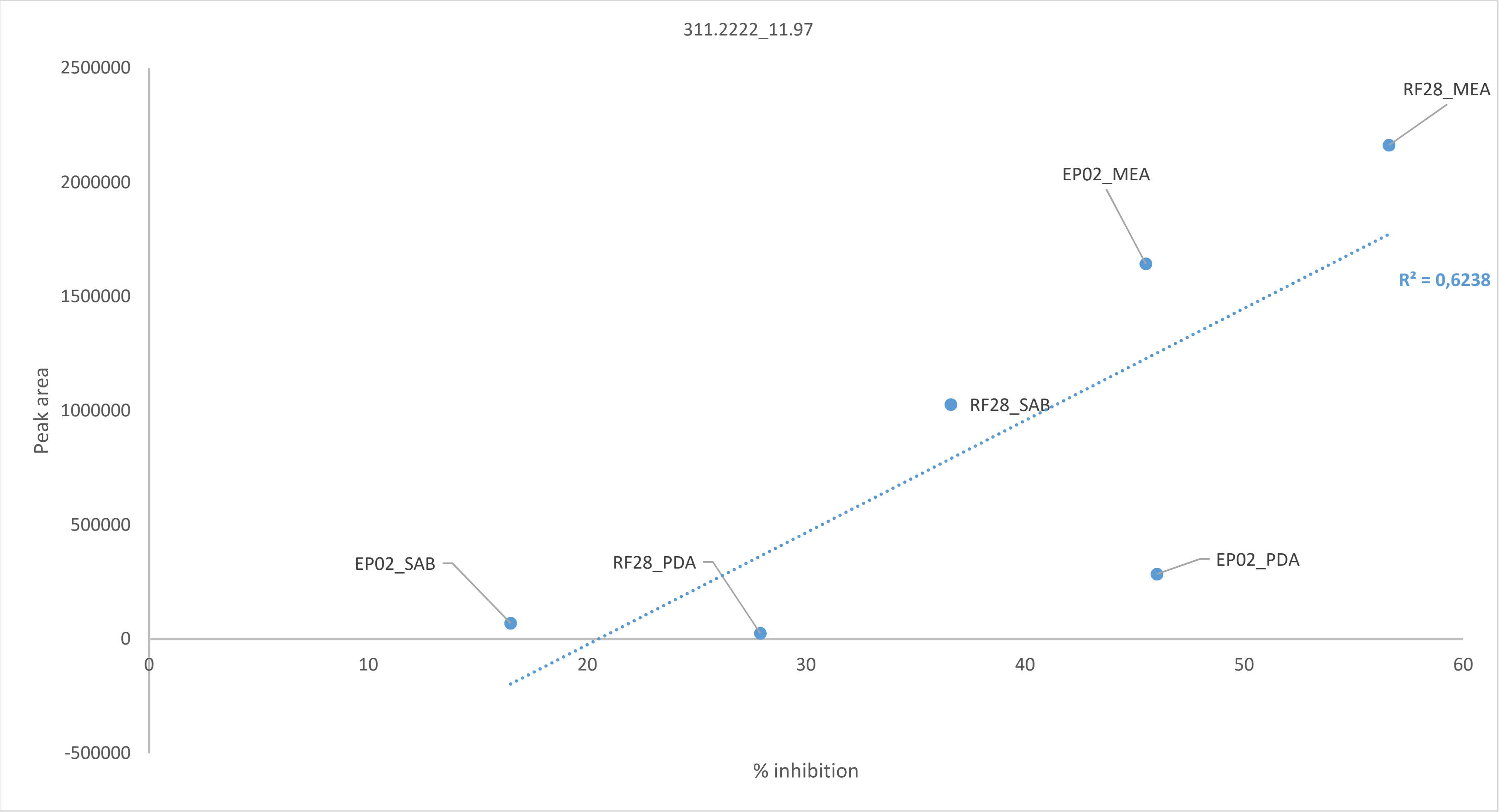

Figure S14. Scatter plot presenting peak areas of highlighted feature *m/z* 295.2274 at 14.6 min in correlation with the activity of the corresponding extracts of *Preussia* samples. Pearson correlation coefficient of the linear regression r² is given.

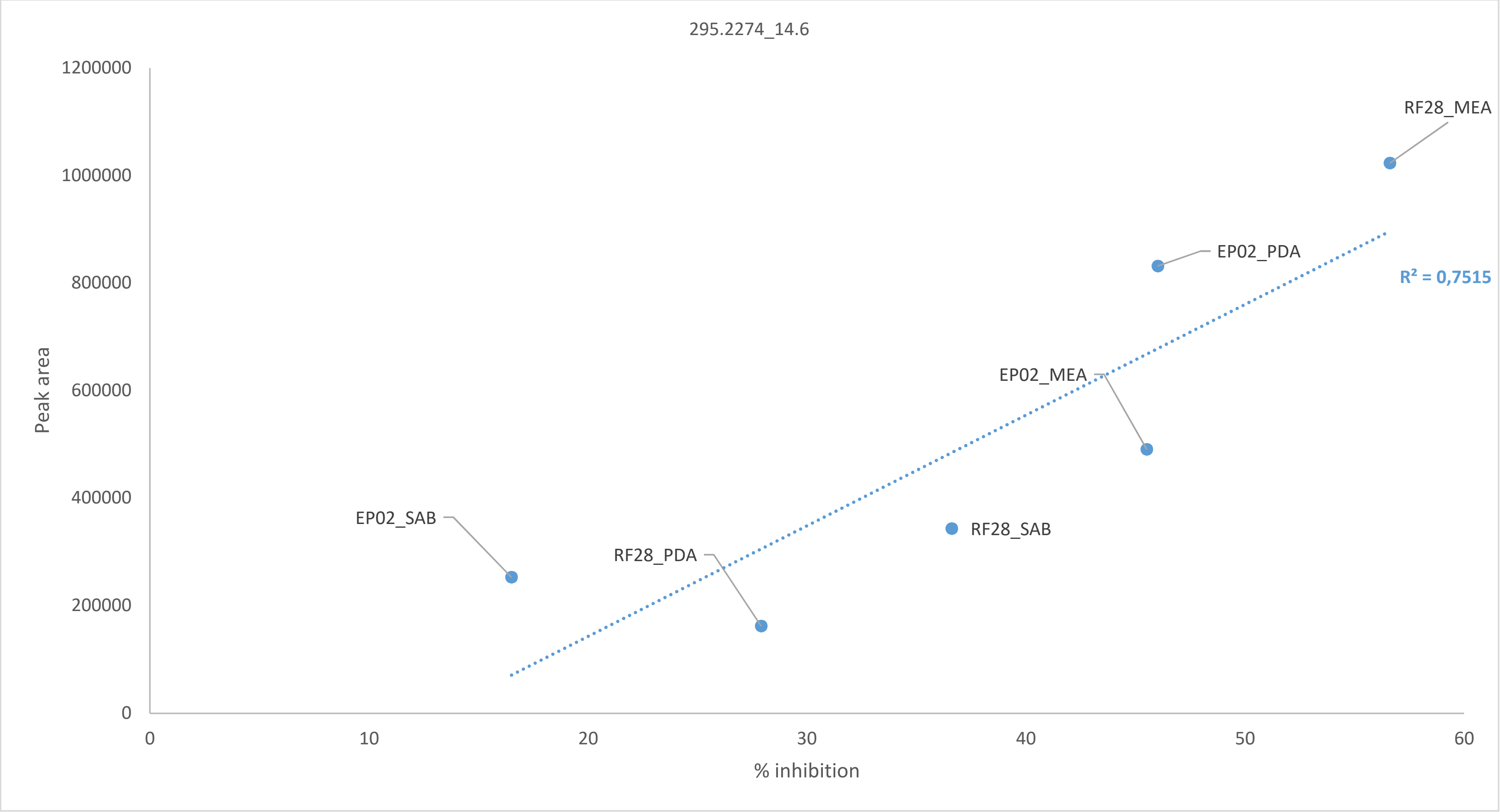

Figure S15. Scatter plot presenting peak areas of highlighted feature *m/z* 2953.2111 at 14.0 min in correlation with the activity of the corresponding extracts of *Preussia* samples. Pearson correlation coefficient of the linear regression r² is given.

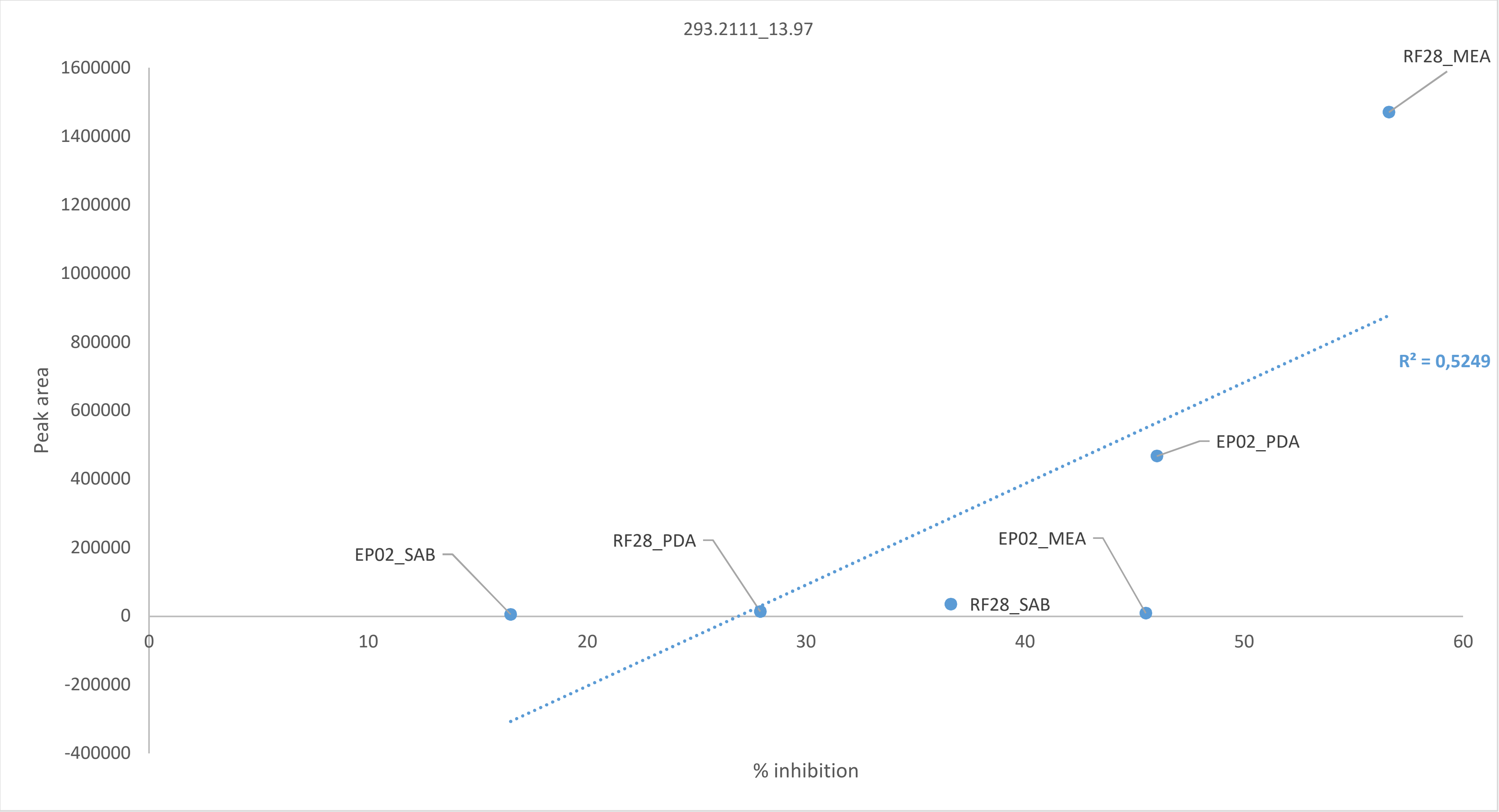

Figure S16. Scatter plot presenting peak areas of highlighted feature *m/z* 591.4622 at 16.5 min in correlation with the activity of the corresponding extracts of *Preussia* samples. Pearson correlation coefficient of the linear regression r² is given.

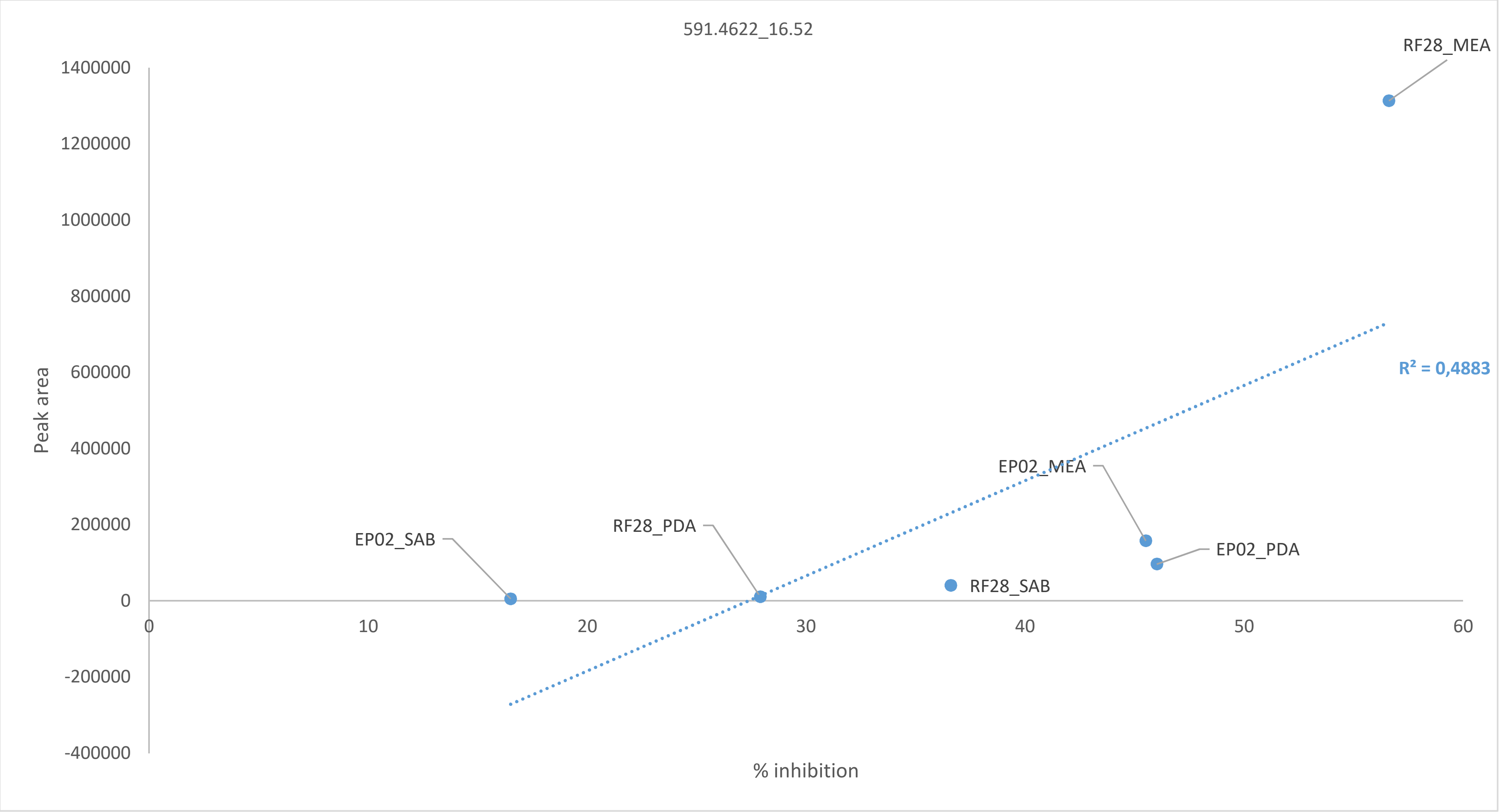

Figure S17. Scatter plot presenting peak areas of highlighted feature *m/z* 298.2115 at 14.2 min in correlation with the activity of the corresponding extracts of *Preussia* samples. Pearson correlation coefficient of the linear regression r² is given.

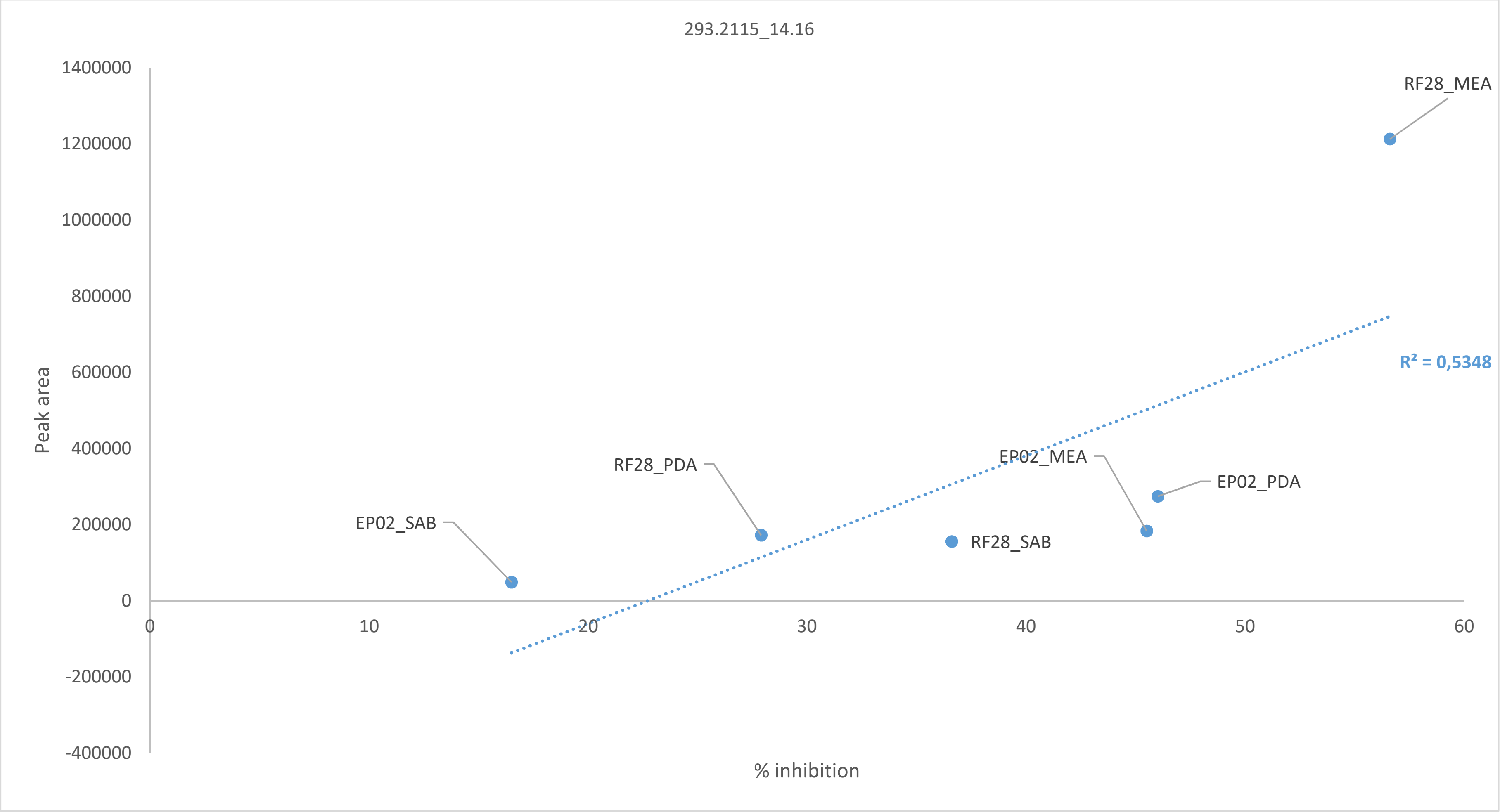

Figure S18. Scatter plot presenting peak areas of highlighted feature *m/z* 307.1907 at 12.9 min in correlation with the activity of the corresponding extracts of *Preussia* samples. Pearson correlation coefficient of the linear regression r² is given.

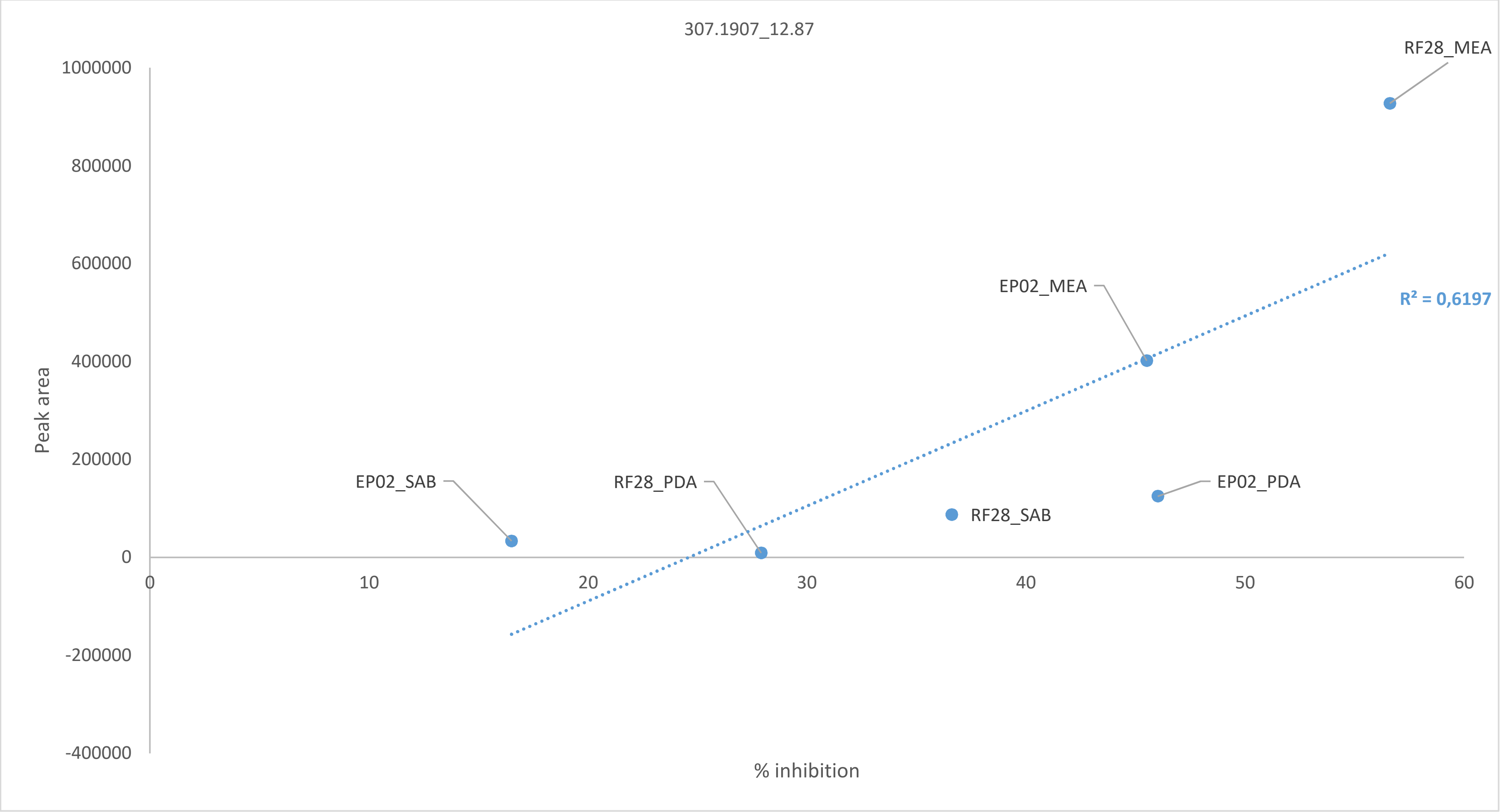

Figure S19. Scatter plot presenting peak areas of highlighted feature *m/z* 311.2217 at 13.9 min in correlation with the activity of the corresponding extracts of *Preussia* samples. Pearson correlation coefficient of the linear regression r² is given.

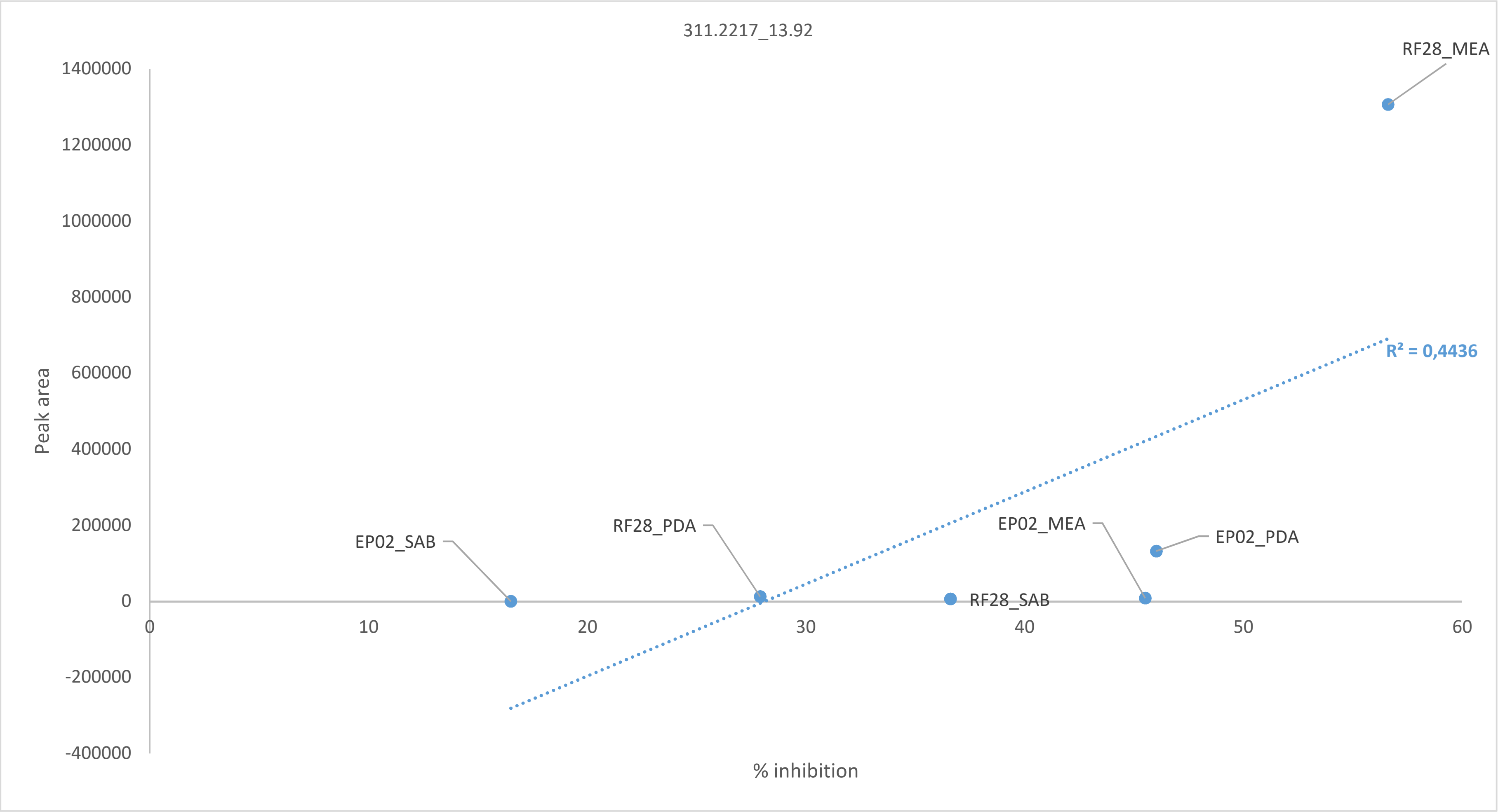

Figure S20. Extracted ion chromatograms for *m/z* 311.2219 for the different extracts of *Preussia* species with cultures on MEA medium highlighted in bold.

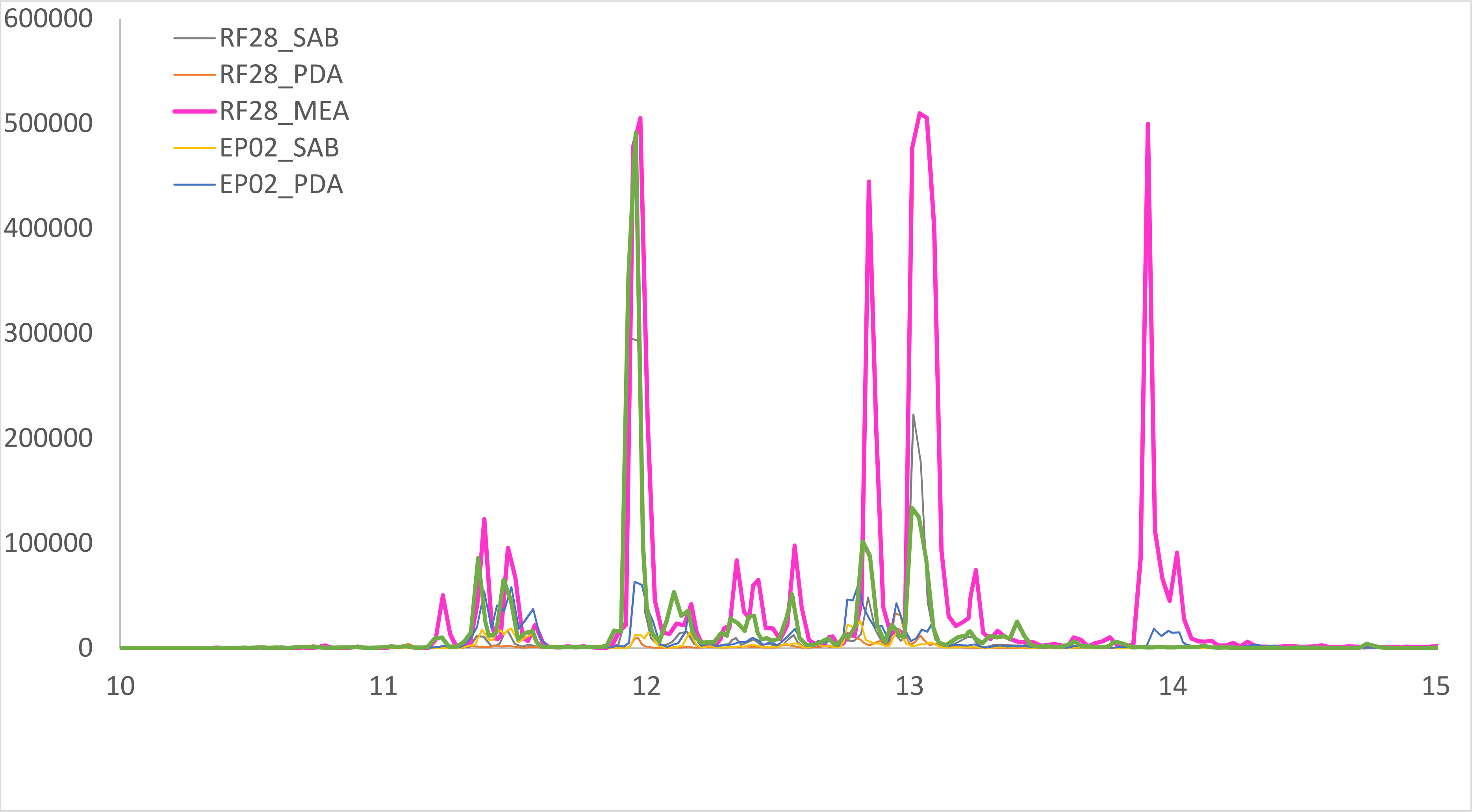

Figure S21. Peak area of the three major peaks for *m/z* 311.222 in the different extracts of *Preussia* species in relation with their culture media : Sabouraud (SAB), Potato Dextrose Agar (PDA), Malt Extract Agar (MEA).

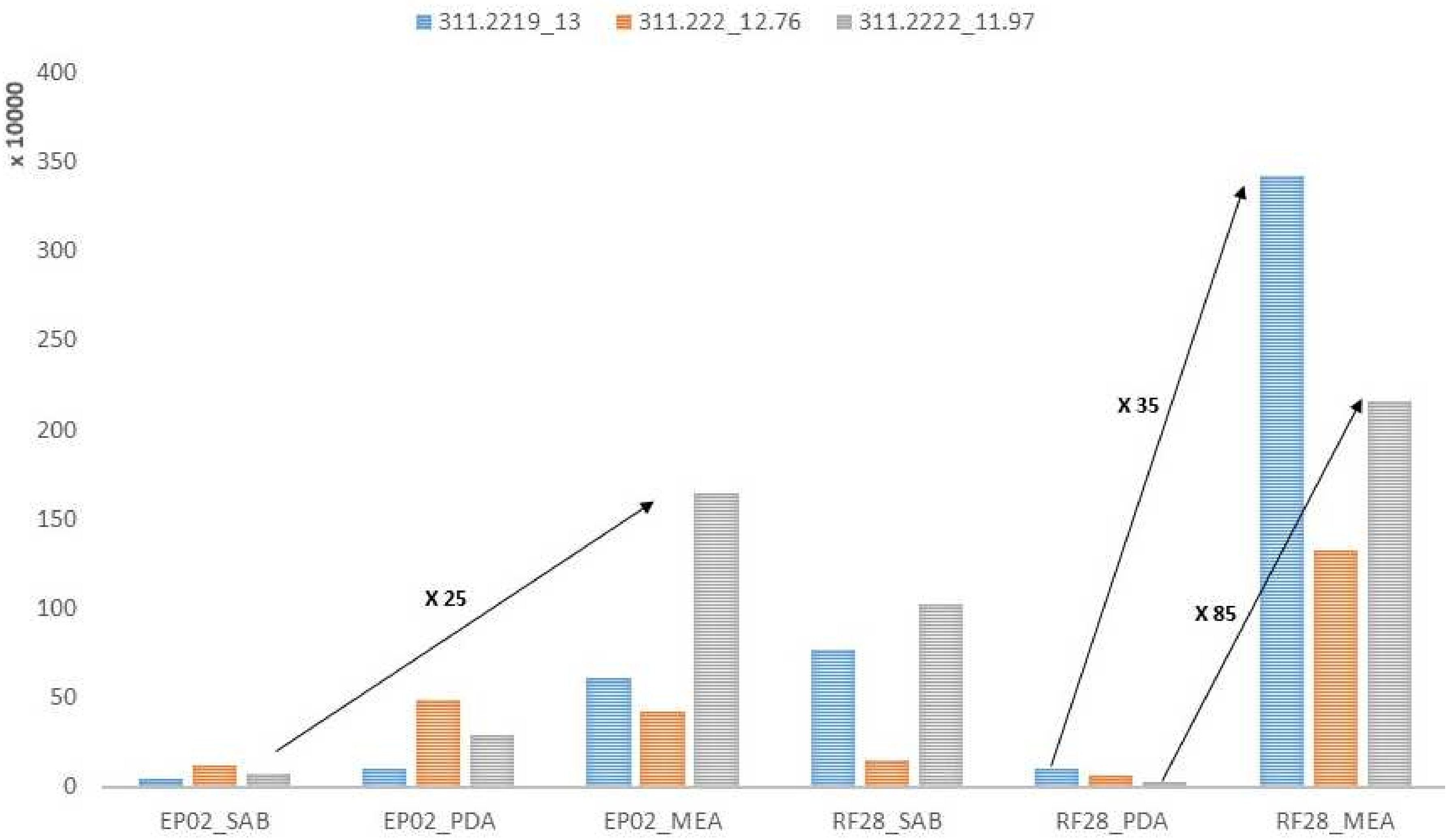

311.2219_13
311.222_12.76
311.2222_11.97
x 10000
400
350
300
250
200
150
100
50
0
X 25
X 35
X 85
EP02_SAB
EP02_PDA
EP02_MEA
RF28_SAB
RF28_PDA
RF28_MEA

Figure S22. 1H NMR of compound 8Z-5,6-epoxy-4-hydroxy-octadec-8-enoic acid

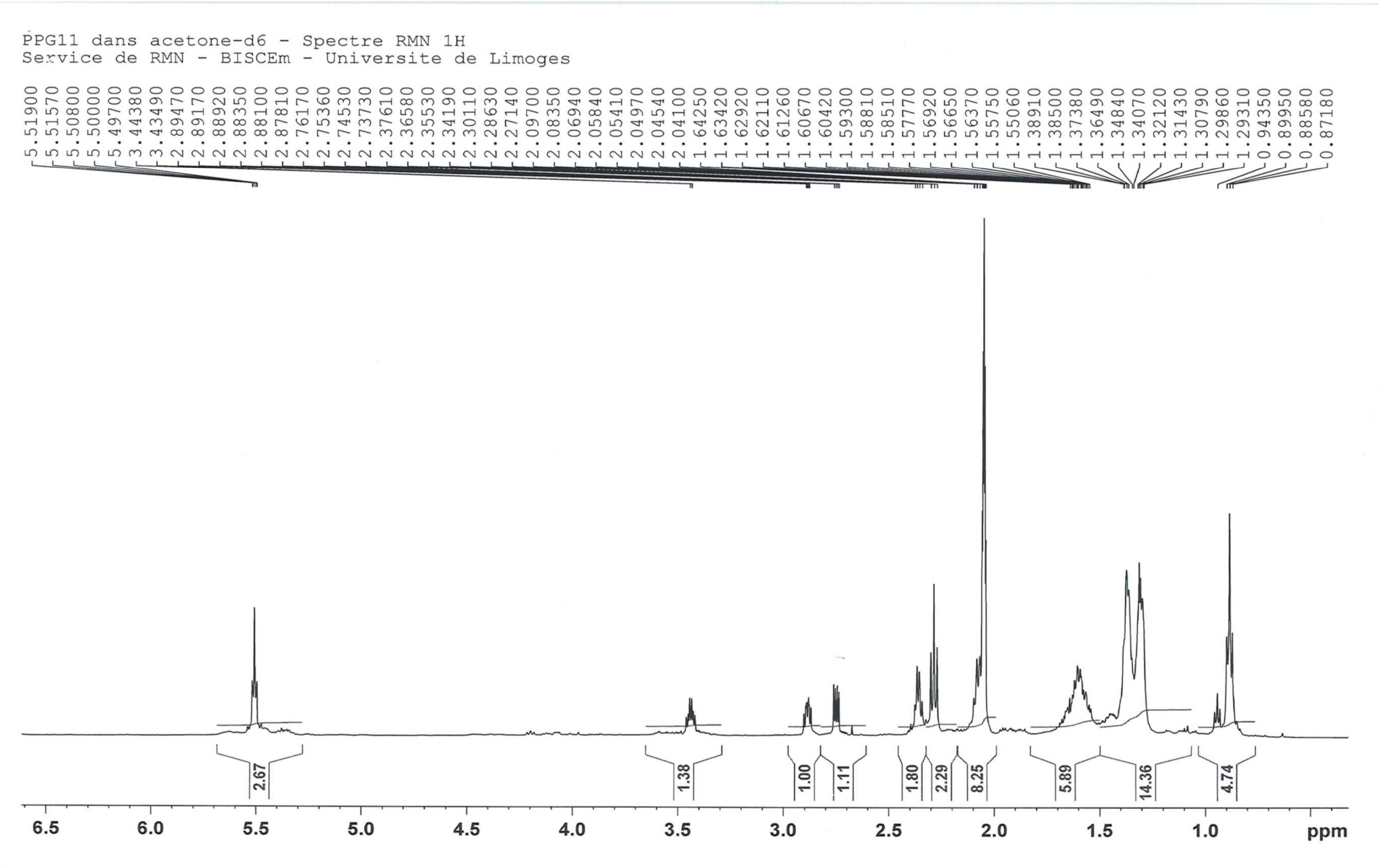

Figure S23. ^{13}C NMR of compound 8Z-5,6-epoxy-4-hydroxy-octadec-8-enoic acid

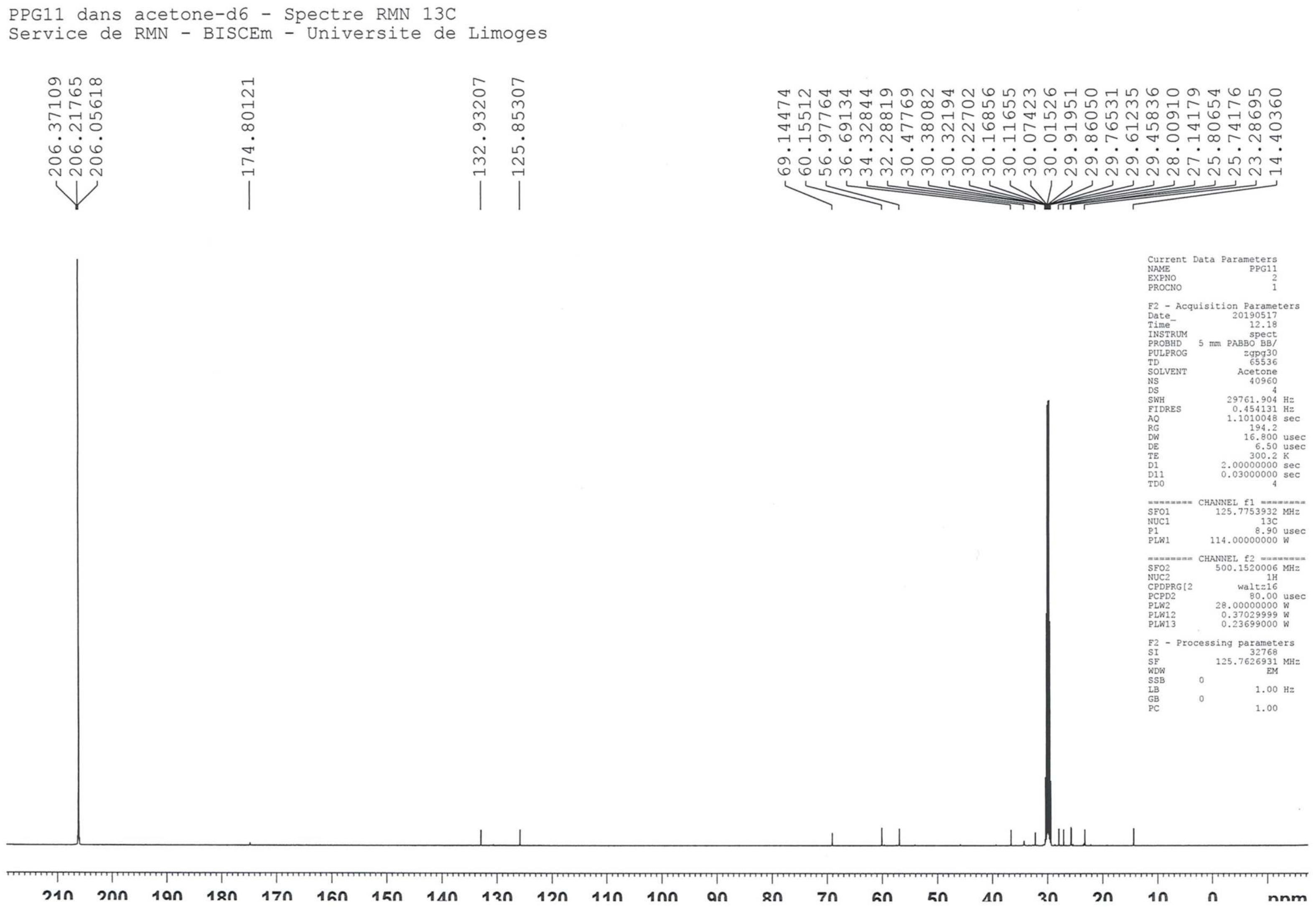

Figure S24. ^{13}C NMR of compound 8Z-5,6-epoxy-4-hydroxy-octadec-8-enoic acid

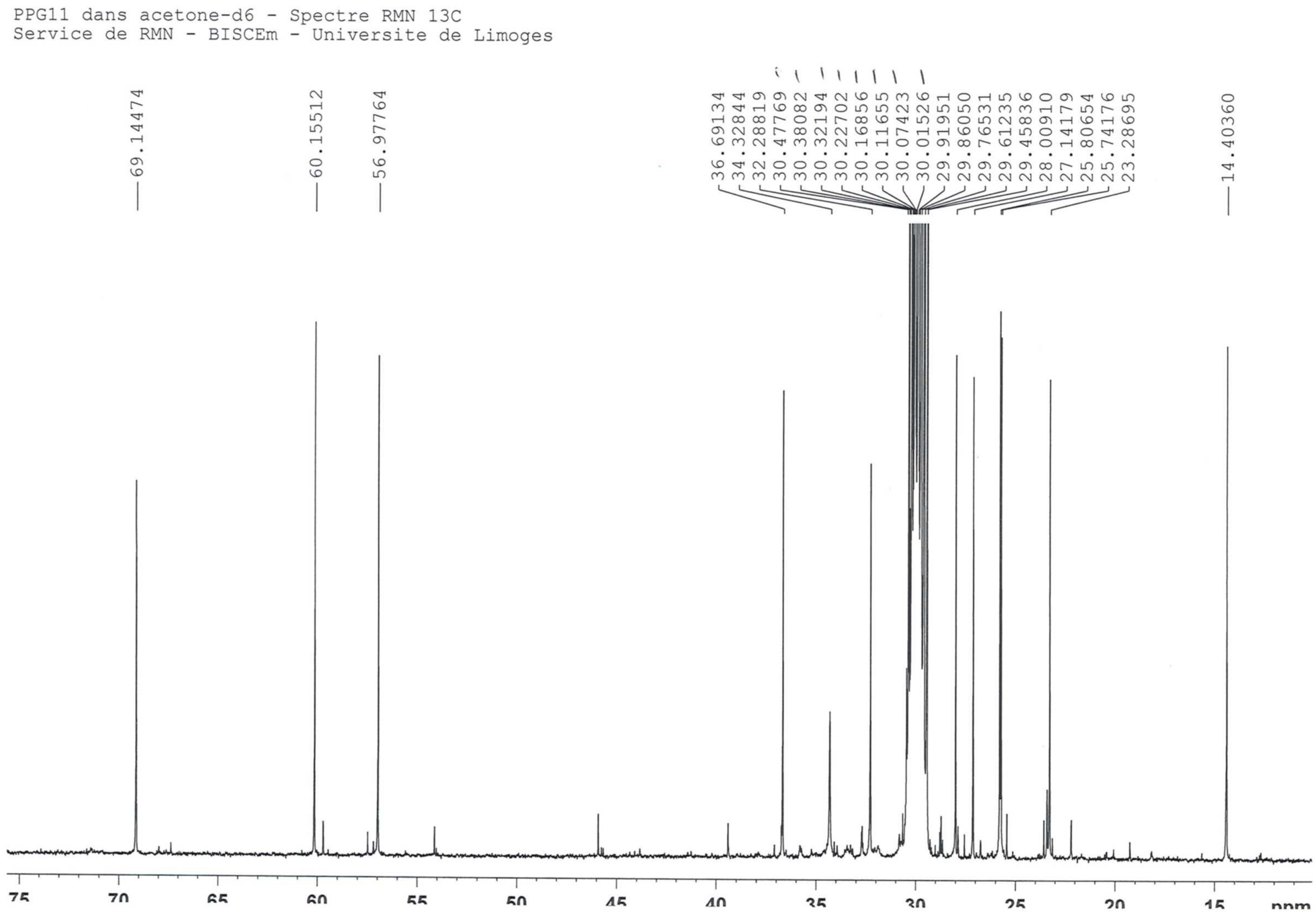

Figure S25. 2D NMR COSY Spectrum of compound 8Z-5,6-epoxy-4-hydroxy-octadec-8-enoic acid

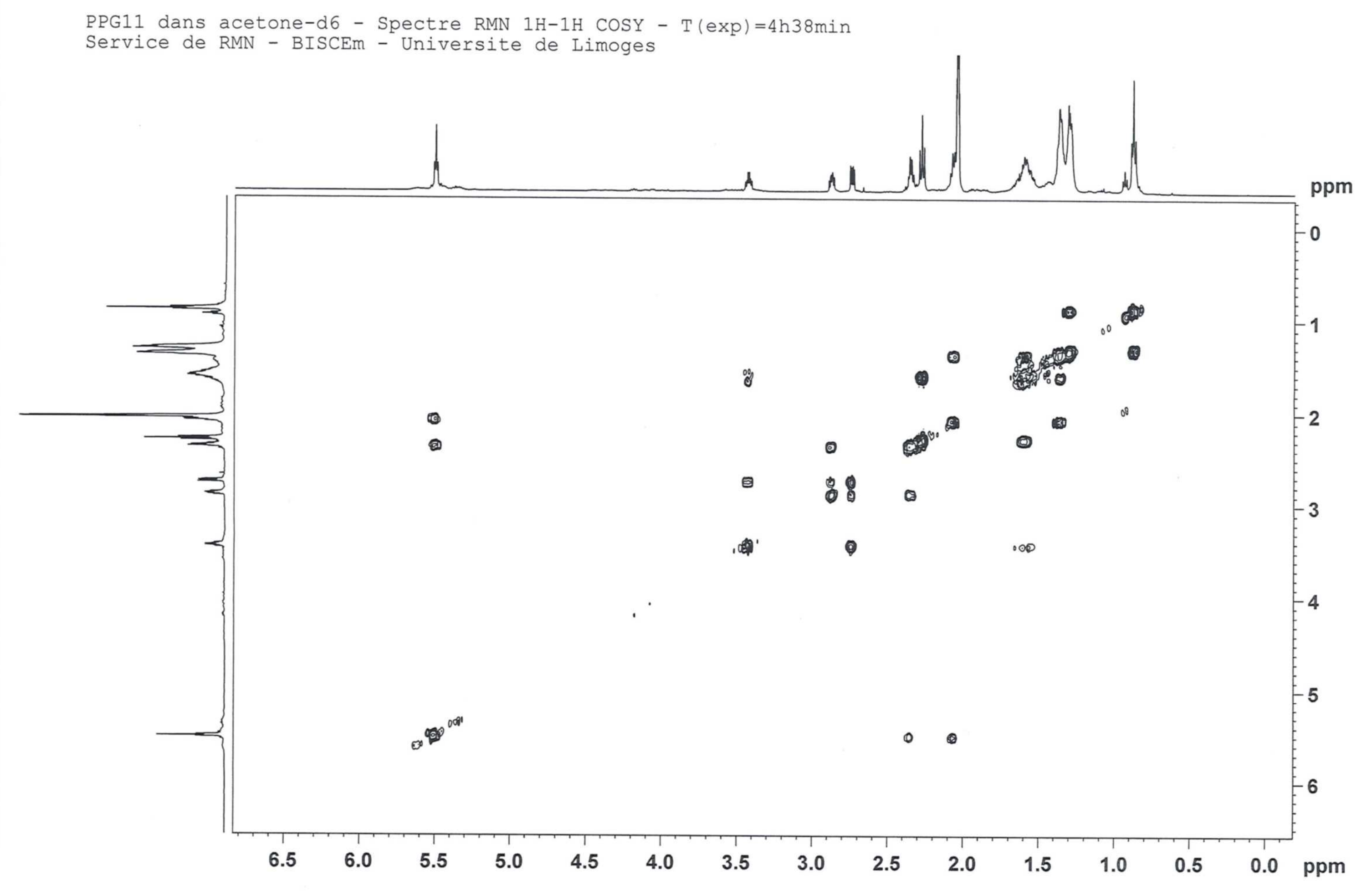

Figure S26. 2D NMR COSY Spectrum of compound 8Z-5,6-epoxy-4-hydroxy-octadec-8-enoic acid

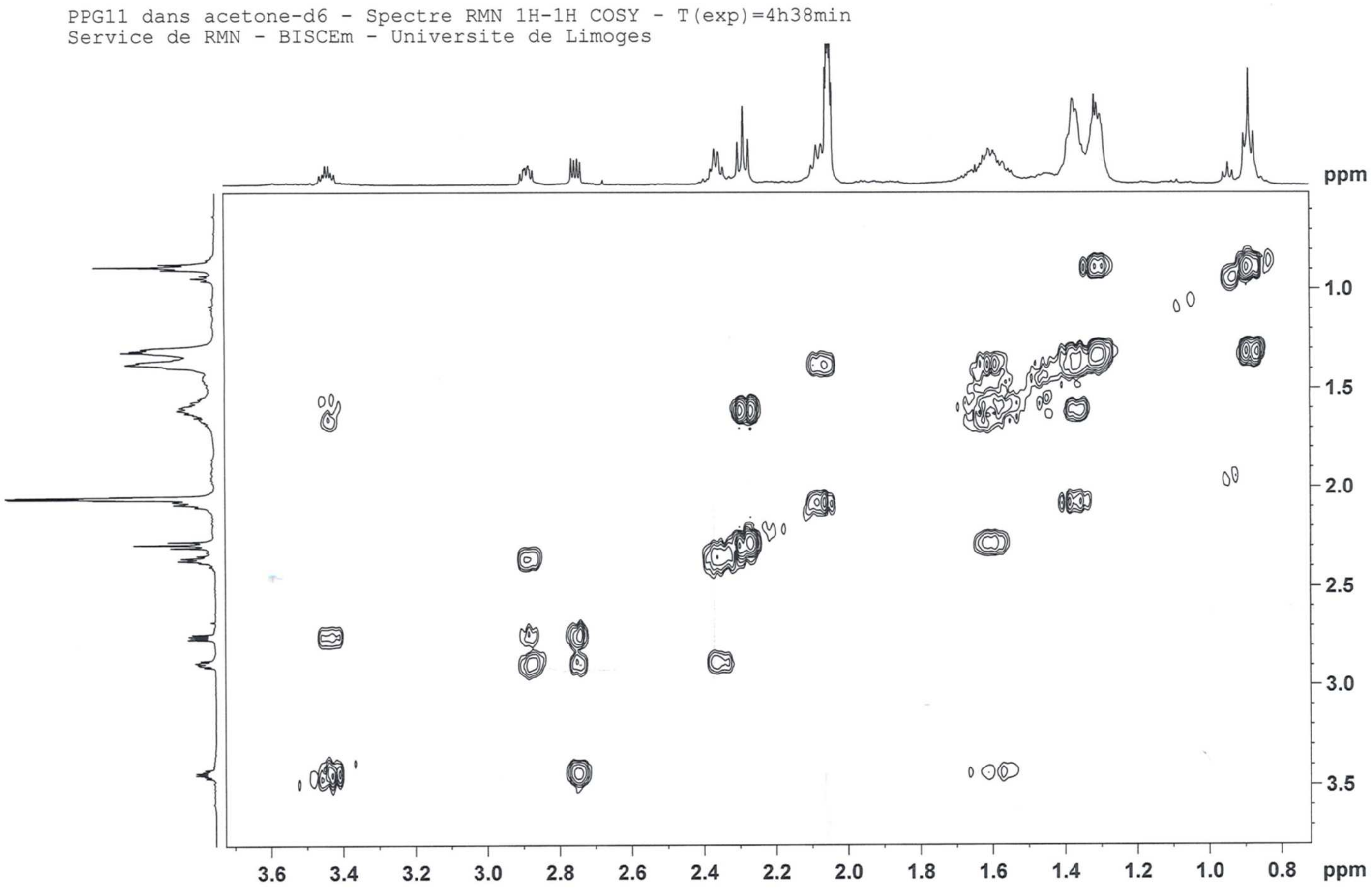

Figure S27. 2D NMR HSQC Spectrum of compound 8Z-5,6-epoxy-4-hydroxy-octadec-8-enoic acid

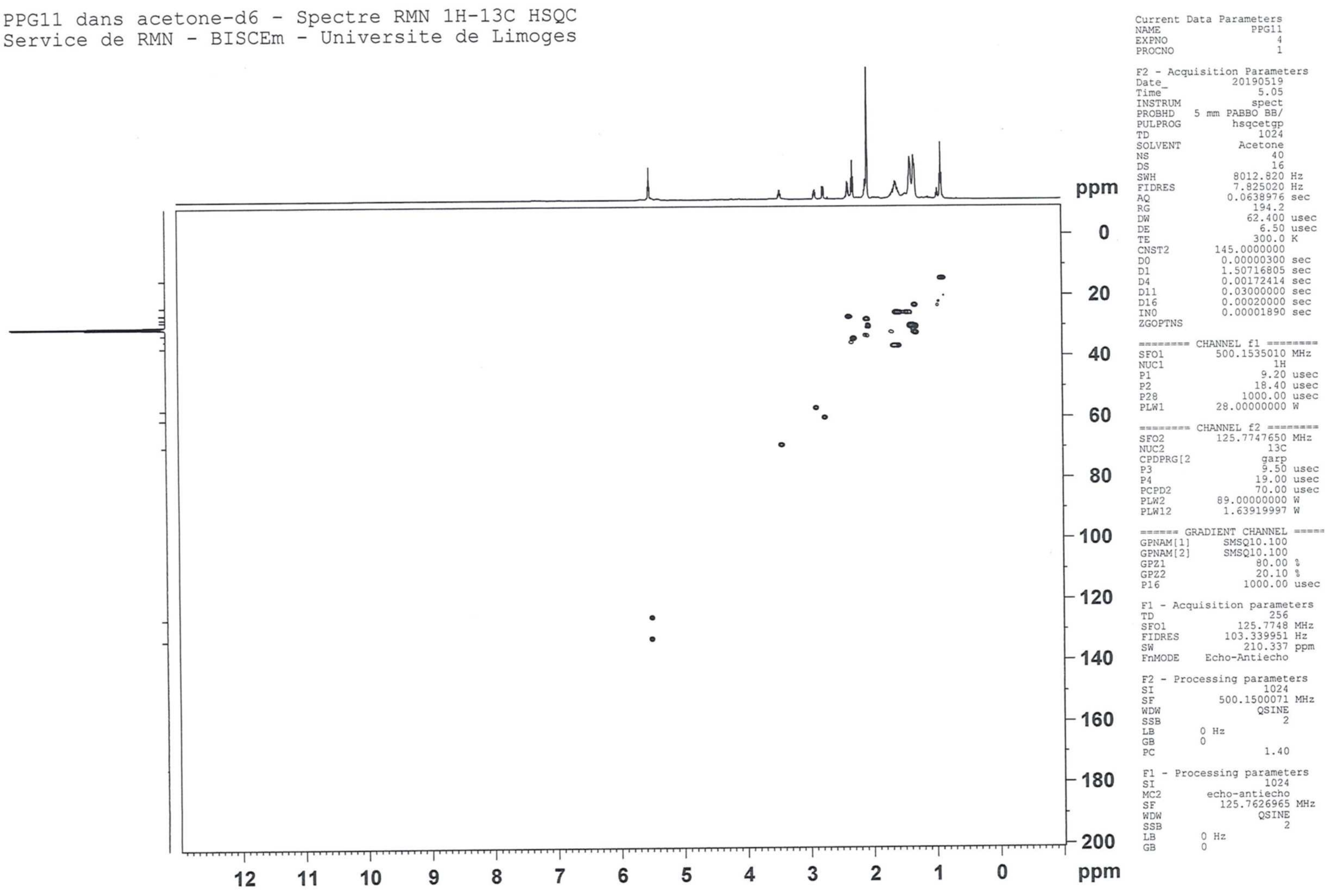

Figure S28. 2D NMR HSQC Spectrum of compound 8Z-5,6-epoxy-4-hydroxy-octadec-8-enoic acid

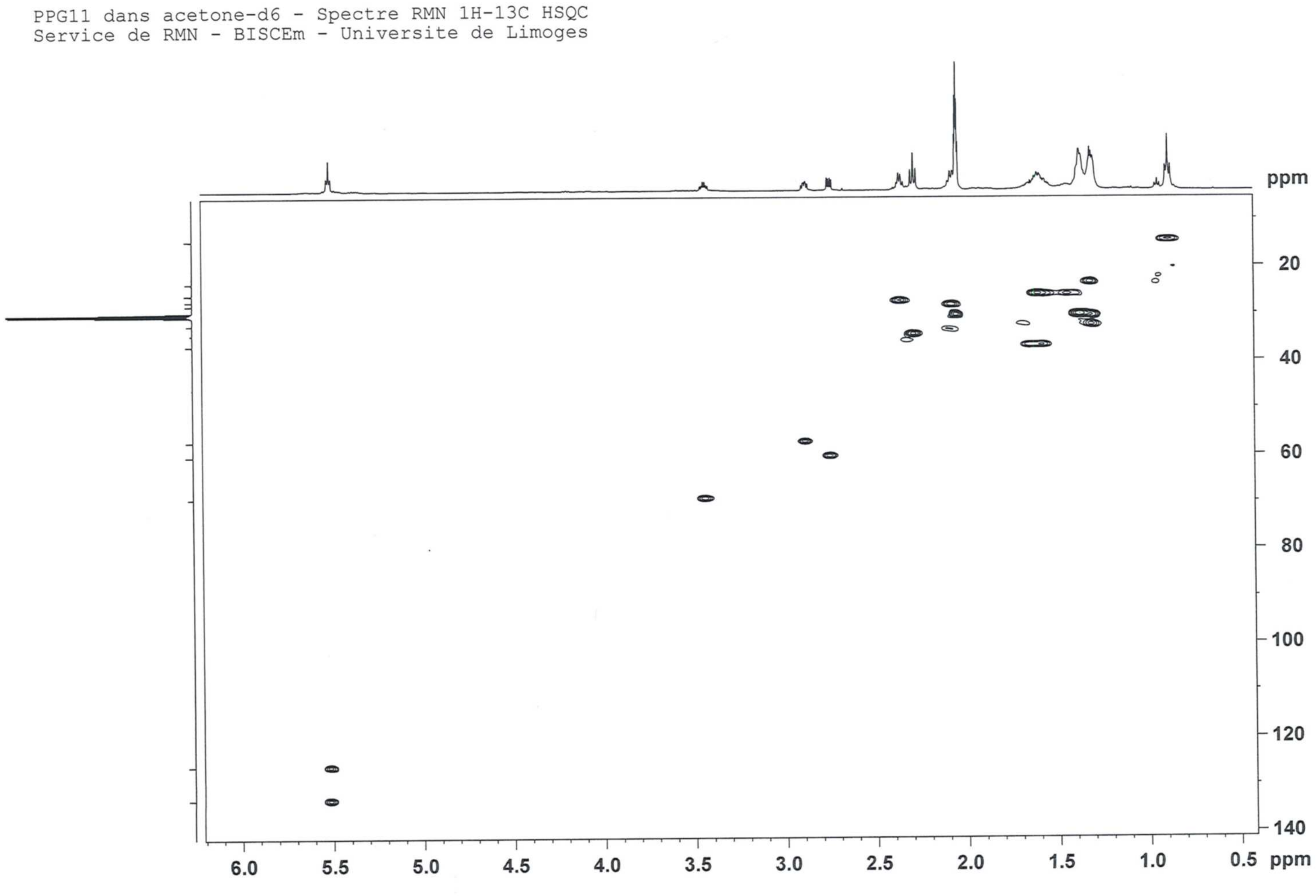

Figure S29. 2D NMR HMBC Spectrum of compound 8Z-5,6-epoxy-4-hydroxy-octadec-8-enoic acid

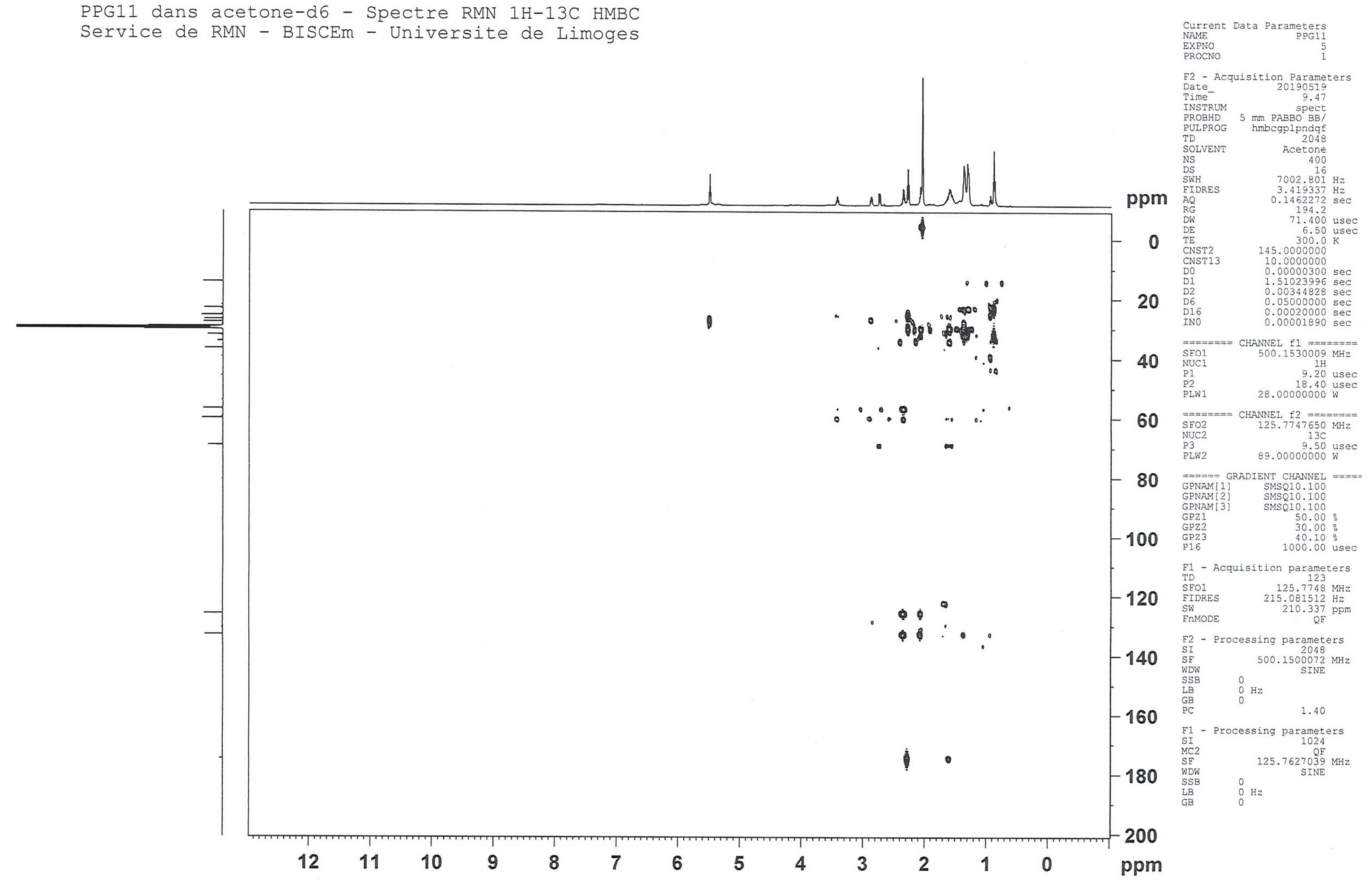

Figure S30. 2D NMR HMBC Spectrum of compound 8Z-5,6-epoxy-4-hydroxy-octadec-8-enoic acid

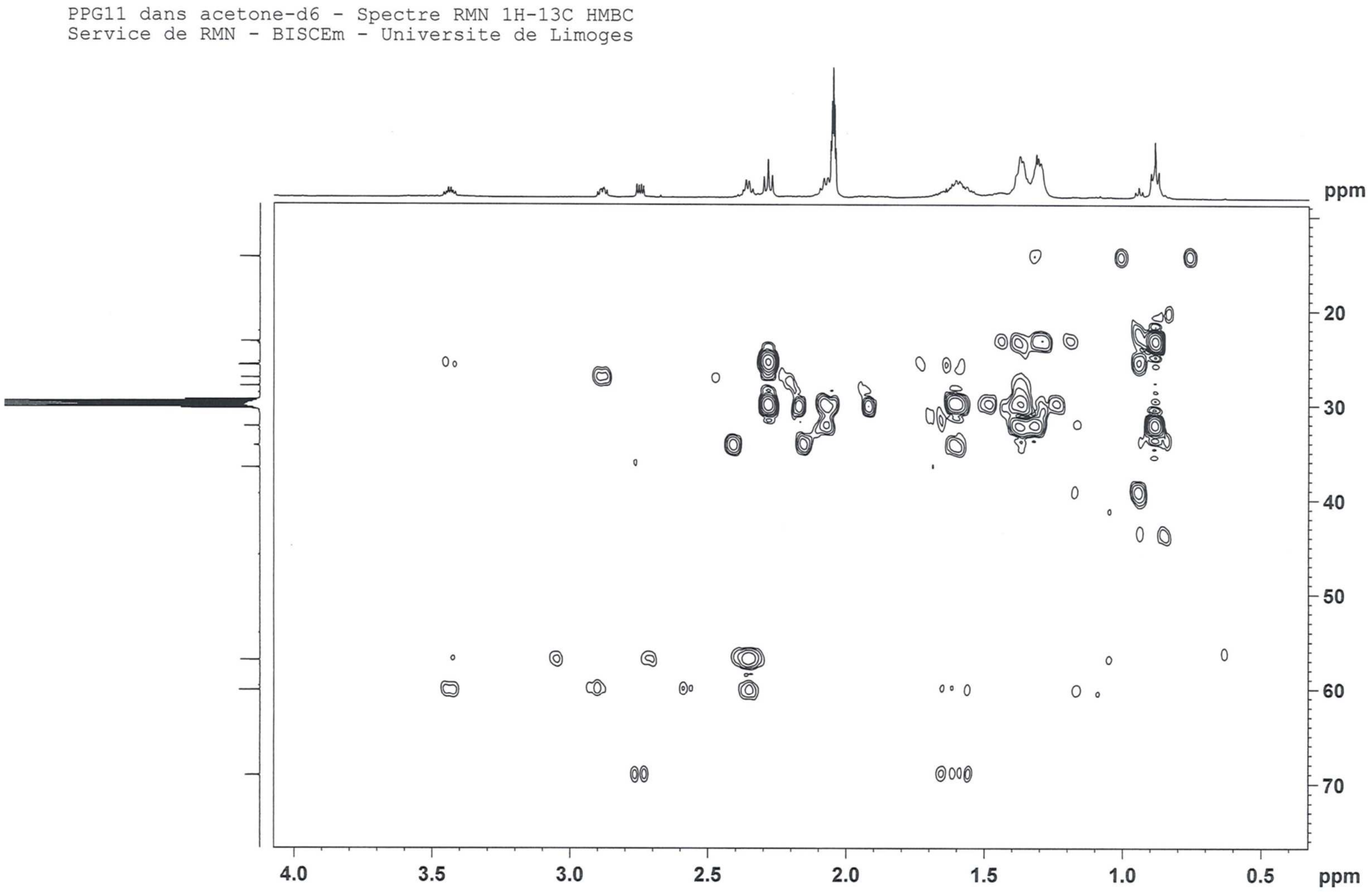

Supplement: Supplementary file 1 [file jof-08-01012-s001.zip › 6-jof-1917199-supplementary revision.pdf]
